# Genetic Variation and Autism: A Field Synopsis and Systematic Meta-Analysis

**DOI:** 10.3390/brainsci10100692

**Published:** 2020-09-30

**Authors:** Jinhee Lee, Min Ji Son, Chei Yun Son, Gwang Hun Jeong, Keum Hwa Lee, Kwang Seob Lee, Younhee Ko, Jong Yeob Kim, Jun Young Lee, Joaquim Radua, Michael Eisenhut, Florence Gressier, Ai Koyanagi, Brendon Stubbs, Marco Solmi, Theodor B. Rais, Andreas Kronbichler, Elena Dragioti, Daniel Fernando Pereira Vasconcelos, Felipe Rodolfo Pereira da Silva, Kalthoum Tizaoui, André Russowsky Brunoni, Andre F. Carvalho, Sarah Cargnin, Salvatore Terrazzino, Andrew Stickley, Lee Smith, Trevor Thompson, Jae Il Shin, Paolo Fusar-Poli

**Affiliations:** 1 Department of Psychiatry, Yonsei University Wonju College of Medicine, Wonju 26426, Korea; jinh.lee95@yonsei.ac.kr; 2 Yonsei University College of Medicine, Seoul 03722, Korea; minji9144@hanmail.net (M.J.S.); crossing96@yonsei.ac.kr (J.Y.K.); 3 Department of Psychological & Brain Sciences, Washington University in St. Louis, St. Louis, MO 63130, USA; hy321321@naver.com; 4 College of Medicine, Gyeongsang National University, Jinju 52727, Korea; gwangh.jeong@gmail.com; 5 Department of Pediatrics, Yonsei University College of Medicine, Seoul 03722, Korea; AZSAGM@yuhs.ac; 6 Severance Hospital, Yonsei University College of Medicine, Seoul 03722, Korea; kwangseob@yuhs.ac; 7 Division of Biomedical Engineering, Hankuk University of Foreign Studies, Gyeonggi-do 17035, Korea; younko@hufs.ac.kr; 8 Department of Nephrology, Yonsei University Wonju College of Medicine, Wonju 26426, Korea; junyoung07@yonsei.ac.kr; 9 Early Psychosis: Interventions and Clinical-detection (EPIC) Lab, Department of Psychosis Studies, Institute of Psychiatry, Psychology & Neuroscience, King’s College London, London SE5 8AB, UK; radua@clinic.cat (J.R.); marco.solmi83@gmail.com (M.S.); 10 Mental Health Networking Biomedical Research Centre (CIBERSAM), 08036 Barcelona, Spain; 11 Centre for Psychiatry Research, Department of Clinical Neuroscience, Karolinska Institute, 11330 Stockholm, Sweden; 12 Institut d’Investigacions Biomèdiques August Pi i Sunyer (IDIBAPS), 08036 Barcelona, Spain; 13 Department of Pediatrics, Luton & Dunstable University Hospital NHS Foundation Trust, Luton LU4ODZ, UK; michael_eisenhut@yahoo.com; 14 CESP, Inserm UMR1178, Department of Psychiatry, Assistance Publique-Hôpitaux de Paris, Bicêtre University Hospital, 94275 Le Kremlin Bicêtre, France; florence.gressier@aphp.fr; 15 Research and Development Unit, Parc Sanitari Sant Joan de Déu, Universitat de Barcelona, Fundació Sant Joan de Déu, CIBERSAM, 08830 Barcelona, Spain; a.koyanagi@pssjd.org; 16 ICREA, Pg. Lluis Companys 23, 08010 Barcelona, Spain; 17 Instituto de Salud Carlos III, Centro de Investigación Biomédica en Red de Salud Mental, CIBERSAM, 28029 Madrid, Spain; 18 Physiotherapy Department, South London and Maudsley NHS Foundation Trust, London SE5 8AZ, UK; brendon.stubbs@kcl.ac.uk; 19 Department of Psychological Medicine, Institute of Psychiatry, Psychology and Neuroscience, King’s College London, London SE5 8AF, UK; 20 Department of Neurosciences, University of Padua, 90133 Padua, Italy; 21 Neurosciences Center, University of Padua, 90133 Padua, Italy; 22 Department of Psychiatry, University of Toledo Medical Center, Toledo, OH 43614, USA; Theodor.Rais@utoledo.edu; 23 Department of Internal Medicine IV, Medical University Innsbruck, Anichstraße 35, 6020 Innsbruck, Austria; andreas.kronbichler@i-med.ac.at; 24 Pain and Rehabilitation Centre, and Department of Health, Medicine and Caring Sciences, Linköping University, SE-581 85 Linköping, Sweden; elena.dragioti@liu.se; 25 Laboratory of Histological Analysis and Preparation (LAPHIS), Federal University of the Parnaiba Delta, Parnaiba 64202-020, Brazil; vasconcelos@ufpi.edu.br (D.F.P.V.); feliperodolfo.15@hotmail.com (F.R.P.d.S.); 26 Department of Basic Sciences, Medicine Faculty of Tunis, Tunis El Manar University, 15 Rue Djebel Lakdar, Tunis 1007, Tunisia; kalttizaoui@gmail.com; 27 University Hospital, University of São Paulo, São Paulo CEP 05508-000, Brazil; brunowsky@gmail.com; 28 Service of Interdisciplinary Neuromodulation, Department and Institute of Psychiatry, University of São Paulo Medical School, São Paulo CEP 01246-903, Brazil; 29 Laboratory of Neuroscience and National Institute of Biomarkers in Neuropsychiatry, Department and Institute of Psychiatry, University of São Paulo Medical School, São Paulo CEP 01246-903, Brazil; 30 Department of Psychiatry and Psychotherapy, University Hospital, LMU Munich, 80336 Munich, Germany; 31 Centre for Addiction & Mental Health, Toronto, ON M6J 1H4, Canada; andre.carvalho@camh.ca; 32 Department of Psychiatry, University of Toronto, Toronto, ON M5T 1R8, Canada; 33 Department of Pharmaceutical Sciences and Interdepartmental Research Center of Pharmacogenetics and Pharmacogenomics (CRIFF), University of Piemonte Orientale, 28100 Novara, Italy; sarah.cargnin@uniupo.it (S.C.); salvatore.terrazzino@uniupo.it (S.T.); 34 The Stockholm Center for Health and Social Change (SCOHOST), Södertörn University, 141 89 Huddinge, Sweden; amstick66@gmail.com; 35 Department of Preventive Intervention for Psychiatric Disorders, National Institute of Mental Health, National Center of Neurology and Psychiatry, 4-1-1 Ogawahigashicho, Kodaira, Tokyo 187-8553, Japan; 36 The Cambridge Centre for Sport and Exercise Sciences, Anglia Ruskin University, Cambridge CB1 1PT, UK; lee.smith@anglia.ac.uk; 37 Department of Psychology, University of Greenwich, London SE10 9LS, UK; T.Thompson@greenwich.ac.uk; 38 OASIS Service, South London and Maudsley NHS Foundation Trust, London SE8 5HA, UK; 39 Department of Brain and Behavioral Sciences, University of Pavia, 27100 Pavia, Italy

**Keywords:** autism spectrum disorder, false positive report probability (FPRP), Bayesian false-discovery probability (BFDP), meta-analysis, Genome-Wide Association Studies (GWAS)

## Abstract

This study aimed to verify noteworthy findings between genetic risk factors and autism spectrum disorder (ASD) by employing the false positive report probability (FPRP) and the Bayesian false-discovery probability (BFDP). PubMed and the Genome-Wide Association Studies (GWAS) catalog were searched from inception to 1 August, 2019. We included meta-analyses on genetic factors of ASD of any study design. Overall, twenty-seven meta-analyses articles from literature searches, and four manually added articles from the GWAS catalog were re-analyzed. This showed that five of 31 comparisons for meta-analyses of observational studies, 40 out of 203 comparisons for the GWAS meta-analyses, and 18 out of 20 comparisons for the GWAS catalog, respectively, had noteworthy estimations under both Bayesian approaches. In this study, we found noteworthy genetic comparisons highly related to an increased risk of ASD. Multiple genetic comparisons were shown to be associated with ASD risk; however, genuine associations should be carefully verified and understood.

## 1. Introduction

Autism spectrum disorder (ASD) is a brain-based neurodevelopmental disorder characterized by pervasive impairments in reciprocal social communication, social interaction, and restricted and repetitive behaviors or interests, resulting in a substantial burden of individuals, families, and society [1,2]. The repeated reports of recent increase in the prevalence of ASD have raised substantial public concerns. For example, in large, nationwide population-based studies, the estimated ASD prevalence was reported to be 2.47% among U.S. children and adolescents in 2014–2016 [3,4,5]. 

Although the full range of etiologies underlying ASD remain largely unexplained, progress has been made in the past decade in identifying some neurobiological and genetic risk factors, and it has been well established that combination of genetic and environmental factors is involved in the etiopathogenesis of autism [1,6]. There is a strong genetic background of ASD, which was demonstrated by the fact that heritability is as high as 80–90% [7,8]. It is possible to estimate the heritability of ASD by taking into the account its covariance within twins, as twins are matched for many characteristics, including in utero and family environment, as well as other developmental aspects [7,9,10].

ASD is polygenic and genetic variants contribute to ASD risk and phenotypic variability. The results of previous studies showed genome-wide genetic links between ASD [11,12]. They indicated that typical variation in social behavior and adaptive functioning and multiple types of genetic risk for ASD influence a continuum of behavioral and developmental traits.

To the best of our knowledge, this is the comprehensive study to summarize the loci that are associated with ASD among the several known loci reported to be related with ASD. We have synthesized all available susceptibility loci for ASD retrieved from meta-analyses regarding the association between the individual polymorphisms and ASD. For the study, we reviewed observational studies, Genome-Wide Association Studies (GWAS) meta-analyses, the combined analysis of GWAS discovery and replication cohorts, the GWAS catalog and GWAS data from GWAS meta-analyses [13]. Furthermore, we applied a Bayesian approaches including false positive report probability (FPRP) and Bayesian false discovery probability (BFDP) to estimate the noteworthiness of the evidence [14,15]. Using these popular Bayesian statistics (i.e., FPRP and BFDP), our study shows that the results of genotype associations between the gene variant and disease were found to be noteworthy (genuine associations). Through these methods, we selected only statistically meaningful values excluding false-positive values and analyzed them again. We aimed to provide an overview to interpret the statistical significance of reported findings and discuss the identified associations in the suggested genetic risk factors for ASD.

## 2. Materials and Methods 

This review was conducted following a registered protocol. The specified methods are available on the PROSPERO database with the registration number CRD42018091704. The Preferred Reporting Items for Systematic Reviews and Meta-Analyses (PRISMA) guidelines of this review are shown in Supplementary Table S1.

### 2.1. Experimental Section

#### 2.1.1. Inclusion and Exclusion Criteria

Studies were included if they satisfied the following conditions: (1) estimated the risk of ASD in humans using meta-analyses in terms of odds ratio (OR) and 95% confidence interval (CI); (2) published in English. Articles were excluded if (1) they did not cover the subject of genetic polymorphism or ASD; (2) did not have individual results for ASD; (3) did not use statistical methods of meta-analysis.

#### 2.1.2. Search Strategy

**Figure 1:**
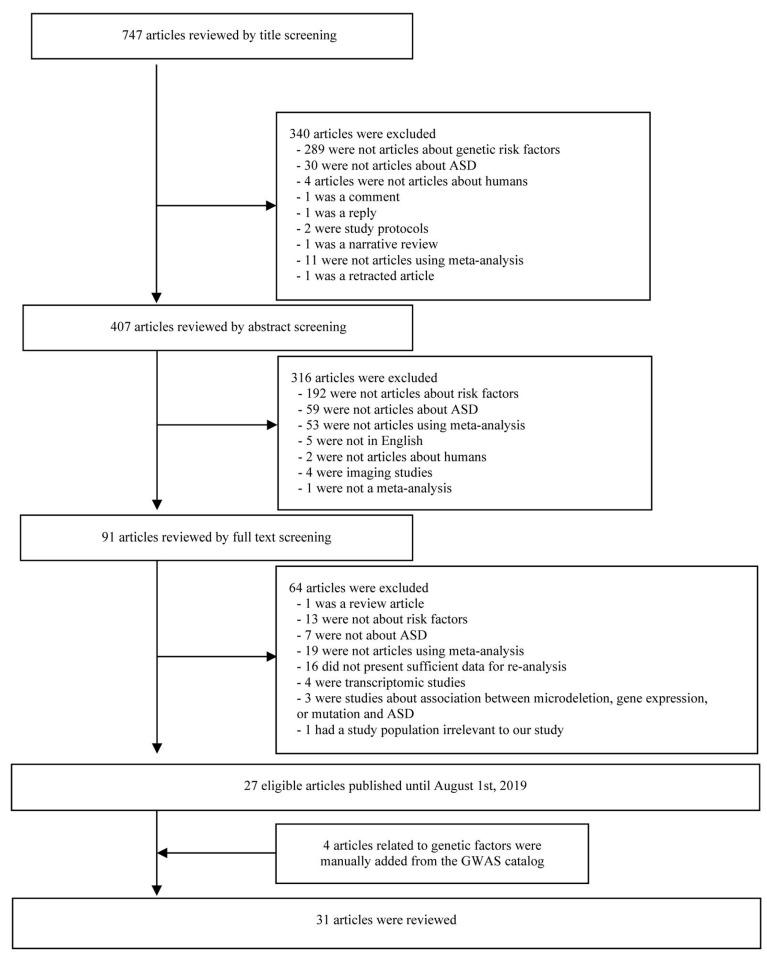
Flow chart of literature search.

A PubMed search was performed to extract data from meta-analyses regarding the gene polymorphisms of ASD published until 1 August, 2019. Two of the authors (MJ Son and CY Son) used the search terms (autism AND meta OR meta-analysis) and obtained relevant articles, first, by scanning the titles and abstracts and, second, by reviewing the full-text (Figure 1). During the selection process, all genetic, gen*, and related terms were included in the relevant articles. Any disagreements were resolved by discussion and consensus. In the case of GWAS, the GWAS catalog was additionally used, as well as PubMed, for a more precise search.

#### 2.1.3. Data Extraction

From each article, we extracted the first author, year of publication, the number of individual studies included, the number of cases and controls, and the number of families if a meta-analysis included family-based studies, the type of statistical model (fixed or random) and study design. We also recorded gene name, gene variants, genotypic comparison, OR with 95% CI, and the corresponding *p*-value. We retrieved all the main data (preferably adjusted), and, for comprehensiveness we additionally extracted subgroup analysis data if the main data were not statistically significant. When data were incomplete, we contacted the corresponding authors for additional information. 

Reported association was considered statistically significant if *p*-value < 0.05 for meta-analyses of observational studies, and <5 × 10^−8^ for GWAS or meta-analyses of GWAS. Meanwhile, genetic associations with a 5 × 10^−8^ < *p*-value < 0.05 were defined as being of borderline significance in GWAS or meta-analyses of GWAS. In addition, we recorded genetic comparisons with *p*-value < 5 × 10^−8^ for our gene network, even when they were not re-analyzable due to insufficient raw data.

### 2.2. Statistical Analysis

Evaluations of the statistical significance of studies about genetic polymorphisms too often inferred false positives, when the evaluations were solely based on *p*-value [15]. Therefore, to clarify “noteworthy” association between re-analyzable genetic variants and ASD, we employed the two Bayesian approaches: FPRP and BFDP [15]. We used the Excel spreadsheets created by Wacholder et al. [15] and Wakefield [14] to calculate FPRP and BFDP, respectively. We computed FPRP at two prior probability levels of 10^−3^ and 10^−6^ and used statistical power to detect two OR levels, 1.2 and 1.5, so that readers can make their own judgment about the evidence for each genetic variant. BFDP is similar to FPRP but uses more information than FPRP [14]. Both prior probability levels were chosen as one of the low and very low values of levels, respectively. We computed BFDP at two prior probabilities levels, 10^-3^ and 10^−6^. We set the thresholds of noteworthiness of FPRP and BFDP to be <0.2 and <0.8, respectively, as recommended by the original papers and highlighted corresponding results in bold type [14,15]. Gene variants were determined to have a noteworthy association with ASD if they satisfied both thresholds.

### 2.3. Construction of Protein-Protein Interaction (PPI) Network

We collected genetic comparisons either with noteworthy results under both FPRP and BFDP or with *p*-value < 5 × 10^−8^ to establish a network of genes using STRING 9.1 (protein-protein interaction network, PPI network) related to ASD [16]. Genetic comparison results, which show genome-wide significance (*p*-value < 5 × 10^−8^) or borderline significance (*p*-value < 0.05) with a noteworthy association under both Bayesian approaches, were included. Any results with a *p*-value < 5 × 10^−8^ that were not re-analyzable were also added in the network analysis. PPI networks provide a critical assessment of protein function on ASD including direct (physical) as well as indirect (functional) associations. 

## 3. Results

### 3.1. Study Characteristics

The initial PubMed literature search yielded 747 articles. Out these, 656 articles were excluded after screening the title and abstract, and 64 articles were omitted after reviewing the full-text. Twenty-seven studies were finally included for the re-analysis of observational studies, GWAS, and meta-analyses of GWAS (Figure 1). 

Additionally, 25 articles were searched on the GWAS catalog, but 14 articles did not meet the criteria were excluded. Among the remaining 11 articles, five articles were not re-analyzable due to insufficient raw data. Moreover, five articles were already included in our dataset from the PubMed search. However, we retained three of the non-re-analyzable articles [17,18,19] since they satisfied the cut-off value of statistical significance for our PPI network (*p*-value < 5 × 10^−8^). Out of the remaining six articles, two were already in our dataset from the literature search from PubMed. Finally, four articles from the GWAS catalog were manually added to 27 articles previously screened from PubMed, leading to a total of 31 eligible articles [17,18,19,20,21,22,23,24,25,26,27,28,29,30,31,32,33,34,35,36,37,38,39,40,41,42,43,44,45,46,47] being included in the systematic review (Figure 1).

### 3.2. Re-Analysis of Meta-Analyses

This paper is divided into two parts: (1) the observational studies part, and (2) the GWAS part. In the observational studies, all statistics were collected considering the overlapping, and results of gene variants with/without statistical significance (Table 1, Supplementary Table S2). Even though genetic variants examined in several studies, we excluded the studies if the data were not significant performed by FPRP or BFDP. In the GWAS part, data from previously published meta-analyses and newly added data from the GWAS catalog were re-analyzed. 

#### 3.2.1. Re-Analysis of Meta-Analyses of Observational Studies 

Among the 31 eligible studies, 19 were meta-analyses of observational studies, which corresponded to 125 genetic comparisons. Thirty one out of 125 genotype comparisons were reported as being statistically significant using the criteria of *p*-value < 0.05 as listed in Table 1.

Out of the 31 genotype comparisons (Table 1), three (9.7%), and two (6.5%) were verified to be noteworthy (<0.2) using FPRP estimation, at a prior probability of 10^−3^ and 10^−6^ with a statistical power to detect an OR of 1.2; seven (22.6%) and two (6.5%) were verified to be noteworthy (<0.2) using FPRP estimation, at a prior probability of 10^−3^ and 10^−6^ with a statistical power to detect an OR of 1.5. In terms of BFDP, five (16.1%) and two (6.5%) comparisons had noteworthy findings (<0.8) at a prior probability of 10^−3^ and 10^−6^. Two single nucleotide polymorphisms (SNPs) were found to be noteworthy under FPRP estimation only, and not under BFDP (Comparison T vs. C, SLC25A12/rs2292813 [20]; C vs. T, SLC25A12/rs2292813 [24]). In contrast, none of the SNPs were identified to be noteworthy exclusively under BFDP. Consequently, five out of 31 SNPs were found noteworthy using both FPRP and BFDP (T vs. C, MTHFR C677T; T (minor), MTHFR C677T; Comparison G vs. A, DRD3/rs167771; C vs. G, RELN/rs362691; A (minor), OXTR/rs7632287).

#### 3.2.2. Re-Analysis of Meta-Analyses of GWAS 

Seven GWAS meta-analyses and one study with a combined analysis of GWAS discovery and replication added up to 203 genetic comparisons [30,31,32,33,34,46,47,48] with statistical or borderline significant results. Out of 277 comparisons, 44 had *p*-value ≥ 0.05 (Table S2), none of which showed noteworthy estimation of FPRP and BFDP with statistical or borderline significant results. From the 203 comparisons, only one (0.5%), MACROD2/rs4141463 A (minor allele), was statistically significant under the genome-wide significance threshold (*p*-value < 5 × 10^−8^), while the remaining 202 comparisons (99.5%) satisfied the criteria of borderline significance (5 × 10^−8^ < *p*-value < 0.05) previously defined.

We examined the 203 genetic comparisons with a genome-wide or borderline significance using both FPRP and BFDP estimation. With FPRP estimation, forty-one (20.2%) and four (2.0%) were assessed to be noteworthy at a prior probability of 10^−3^ and 10^−6^ with statistical power to detect an OR of 1.2. Moreover, fifty-four (26.6%) and eight (3.9%) were identified as noteworthy at a prior probability of 10^−3^ and 10^−6^ with statistical power to detect an OR of 1.5. Overall, forty genetic comparisons (19.7%) were found noteworthy under both Bayesian approaches, which included a single genetic comparison satisfying the conventional significance threshold of *p*-value < 0.05 (Table 2).

#### 3.2.3. Re-Analysis of Results from the GWAS Catalog and GWAS Datasets Included in the GWAS Meta-Analyses 

Genetic comparisons additionally extracted from the GWAS catalog were also re-analyzed (Table 3). Among the 20 included comparisons, two (10.0%) genotype comparisons, MACROD2/rs4141463 and LOCI105370358-LOCI107984602/rs4773054, extracted from the GWAS catalog were reported to be significant with a *p*-value < 5 × 10^−8^. The remaining 18 comparisons were of borderline statistical significance (*p*-value between 0.05 and 5 × 10^−8^). 

While assessing noteworthiness, five (25.0%) and three (15.0%) were verified as being noteworthy using FPRP estimation, at a prior probability of 10^−3^ and 10^−6^, respectively, with the statistical power to detect a 1.2 OR. In addition, eighteen (90.0%) and four (25.0%) showed noteworthiness at a prior probability of 10^−3^ and 10^−6^ with the statistical power to detect a 1.5 OR, respectively. In the BFDP estimation, nineteen (95.0%) and two (10.0%) were assessed as being noteworthy at a prior probability of 10^−3^ and 10^−6^, respectively. Finally, 18 genetic associations (95%) of both significant and borderline statistically significant results were verified as being noteworthy under both the FPRP and BFDP approaches. The total number of associations included two comparisons with genome-wide significance (*p*-value < 5 × 10^−8^) and sixteen comparisons with borderline significance (*p*-value between 0.05 and 5 × 10^−8^).

In order to develop the analysis further, we extracted the GWAS data that was both statistically significant and noteworthy under both Bayesian approaches, from the GWAS meta-analysis and GWAS catalog. They were extracted from five articles [30,31,32,33,34], with 70 of the GWAS data being noteworthy under both FPRP and BFDP. Results with noteworthy association are summarized in Table 4.

### 3.3. Protein-Protein Interaction (PPI) Network 

We established PPI networks related to the risk of ASD by filtering genes noteworthy under both FPRP and BFDP or genes with a *p*-value < 5 × 10^−8^. We included the results of both re-analyzed and non-re-analyzable genetic comparisons from meta-analyses of observational studies and GWAS, GWAS included in meta-analyses of GWAS, and the GWAS catalog. The statistically significant results of non-re-analyzable studies are presented in the Supplement Table S3.

**Figure 2:**
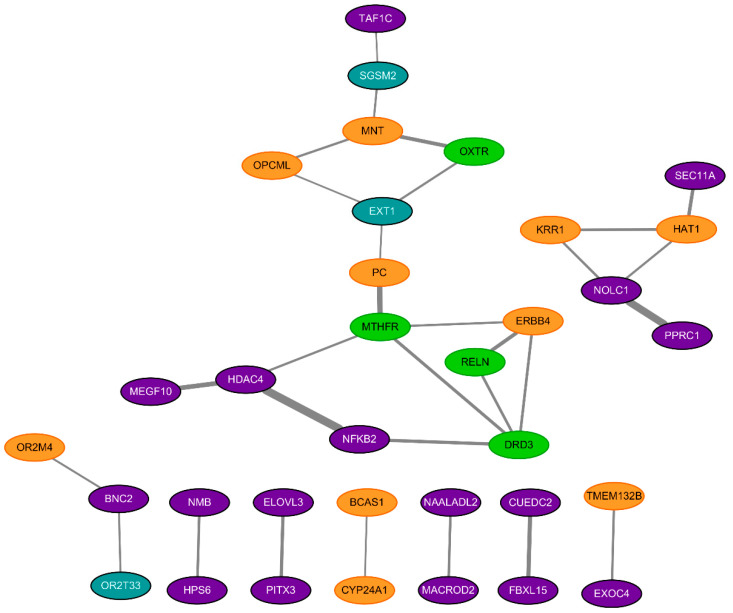
Protein-protein interaction network of ASD. There were 34 distinct genes with about 30 genetic connections among them. The thickness of the line connecting genes represents the score of PPI interaction using STRING9.1 and the color of each gene represents the source of the data; orange, GWAS data: green, GWAS catalog: purple, meta-analysis of GWAS: light green, meta-analysis of observational studies.

The major genes that included a strong genetic connection were the myc-associated factor X (MAX) network transcriptional repressor (MNT), oxytocin receptor (OXTR), nucleolar and coiled-body phosphoprotein (NOLC1), peroxisome proliferator-activated receptor gamma related coactivator-related 1 (PPRC1), pyruvate carboxylase (PC), methylenetetrahydrofolate reductase (MTHFR), multiple epidermal growth factor like domains 10 (MEGF10), nuclear factor kappa B subunit 2 (NFKB2), histone deacetylase 4 (HDAC4), etc. (Figure 2 and Table 5).

## 4. Discussion

To our knowledge, this study is the first study of ASD genetic risk factors, which assessed the levels of evidence of the published meta-analyses showing the association between susceptible loci and ASD. Overall, genetic comparisons with noteworthy results were confirmed as risk factors for ASD. The genetic comparisons highly related to an increased risk of ASD might reflect the implication in neurodevelopment and specific synaptogenesis of ASD. 

According to the PPI network, composed of noteworthy results obtained when using both Bayesian approaches, multiple genes were included as a risk factor for ASD. Investigating the lists genes as a risk factor, promising candidates encoded the protein associated with neural development and specification, and also with neurotransmitters and its receptors. These genes were RELN and DRD3 from observational studies, and PC, OPCML, ERBB4, OR2M4, MEGF10, OR2T33, NMB, and NOLC1, from GWAS. In line with our findings, previous reports have supported that the migration and proliferation of neuronal cells is essential to understanding neurodevelopmental disorders such as ASD or schizophrenia [49,50]. In addition, apart from anatomical approaches, genes correlated with neuropeptides and receptors, such as those in the brain or hippocampus, also explain the pathophysiology of the disease at a molecular level [51]. The list of genes included is presented in Table 5.

The present comprehensive re-analyses shows that, although a large number of studies have suggested numerous possible genetic risk factors for ASD, truly significant results are small and a partial part of whole results. For instance, we detected false positive results in 26 out of 31 (83.9%) meta-analyses of observational studies and 163 out of 203 (80.3%) in meta-analyses of GWAS, respectively. However, only a small portion of genetic comparisons with a *p*-value < 0.05 exhibited noteworthy associations with ASD under both Bayesian approaches (Table 1, Table 2, Table 3 and Table 4).

Moreover, we also detected that genetic comparisons with borderline statistical significance (5 × 10^−8^ < *p*-value < 0.05) accounted for 53 out of 126 (42%) noteworthy comparisons from GWAS or meta-analyses of GWAS. These genetic comparisons might have been neglected if the *p*-value alone was considered to determine noteworthiness. Using the two Bayesian approaches as we did, or relaxing the current GWAS threshold as Panagiotou et al. suggests, might enable better interpretation of GWAS results [48].

Based on the observational studies, out of 31 statistically significant genotype comparisons, five (16.1%) were found noteworthy under both FPRP and BFDP: T vs. C, MTHFR C677T; T (minor), MTHFR C677T; G vs. A, DRD3/rs167771; C vs. G, RELN/rs362691; A (minor), OXTR/rs7632287. From the meta-analyses of GWAS, we could confirm that 34 distinct genes are noteworthy under both Bayesian approaches with about 30 genetic connections. However, the fact that all three comparisons with a *p*-value < 5 × 10^−8^—rs1879532 (Table S3), rs4773054 (Table 2), rs4141463 (Table 2)—displayed noteworthiness may indicate that the stringent threshold of *p* < 5 × 10^−8^ is a good tool for verification of the true noteworthiness of genetic risk factors.

There are several limitations in our review. First, we did not include studies that have not been meta-analyzed, or meta-analyses that had insufficient data in our review. Secondly, we only included the single findings of a meta-analysis with the lowest *p*-value per genetic variant. Therefore, we could not consider potentially meaningful subgroup analyses for different ethnicity, location, gender, and type of genotype comparison (i.e., random or fixed) when selecting a certain outcome. We focused on whether the individual genotype variant was truly associated with ASD or not, regardless of the specific type of the genotype comparison or ethnicity.

Our study has several strengths and implications. For example, to our knowledge, this is the first study that simultaneously analyzed a sizeable amount of data about genetic factors including not only GWAS but also the GWAS catalog. Despite the known high heritability of ASD and abundant research in ASD that has focused on the underlying genetic causes, the literature on genetic risk factors for ASD has not fully reached a consensus. This comprehensive review of genetic associations linked to ASD may improve understanding of the strengths and limitations of each form of research, and advance better and novel approaches for examining ASD in the field of genetic research. The findings of this study could provide mechanisms that may be explored for the development of novel neurotherapeutic agents both for the prevention and treatment of ASD.

## 5. Conclusions

In conclusion, we synthesized published meta-analyses on risk factors of ASD to acquire noteworthy findings and false positive results by adopting two Bayesian approaches for genetic factors. We attempted to synthesize all meta-analyses on genetic polymorphisms linked to ASD and found noteworthy genetic factors highly related to an increased risk of ASD. We also investigated their validity by discovering false positive results under Bayesian methods. To verify results obtained from genetic analyses, both approaches may have advantages, especially for interpretation of results obtained from observational studies. We found noteworthy results from GWAS, not only with *p*-value ranging between 0.05 and 5 × 10^−8^, but also from genetic variants within borderline significance rage which were almost half of the genetic variants. This finding speculates that the genetic variants with borderline significance needs to be further analyzed to determine what associations are genuine. 

## Figures and Tables

**Table 1:** Re-analysis results of gene variants with statistical significance (*p*-value < 0.05) from observational studies.

Author, Year	Gene/Variant	Comparison	OR (95% CI)	*p*-Value	Model	No. of Studies	Power OR 1.2	Power OR 1.5	FPRP Values at Prior Probability				BFDP 0.001	BFDP 0.000001
									OR 1.2		OR 1.5			
									0.001	0.000001	0.001	0.000001		
*Gene variants with statistically significance (p-value < 0.05), FPRP < 0.2 and BFDP < 0.8 from observational studies*														
Rai 2016 [21]	MTHFR C677T	T vs. C	1.37 (1.25, 1.50)	<0.0001	Fixed	Overall (13)	0.002	0.975	0.000	0.005	0.000	0.000	0.000	0.001
Mohammad et al., 2016 [23]	MTHFR C677T	T (minor)	1.47 (1.31, 1.65)	<0.0001	Fixed	Overall (8)	0.000	0.634	0.000	0.179	0.000	0.000	0.000	0.009
Warrier et al., 2015 [24]	DRD3/rs167771	G vs. A	1.822 (1.398, 2.375)	9.08 × 10^−6^	Fixed	Overall (2)	0.001	0.075	0.901	1.000	0.108	0.992	0.649	0.999
Warrier et al., 2015 [24]	RELN/rs362691	C vs. G	0.832 (0.763, 0.908)	3.93 × 10^−5^	Fixed	Overall (6)	0.486	1.000	0.071	0.987	0.036	0.974	0.584	0.999
LoParo et al., 2015 [26]	OXTR/rs7632287	A (minor)	1.43 (1.23, 1.68)	0.000005	Random	Caucasian (2)	0.016	0.720	0.451	0.999	0.018	0.950	0.432	0.999
*Gene variants with statistically significance (p-value < 0.05), FPRP > 0.2 or BFDP > 0.8 from observational studies*														
Liu et al., 2015 [20]	SLC25A12/rs2056202	T vs. C	0.809 (0.713, 0.917)	0.001	Fixed	Overall (8)	0.321	0.999	0.740	1.000	0.478	0.999	0.957	1.000
Liu et al., 2015 [20]	SLC25A12/rs2292813	T vs. C	0.752 (0.649,0.871)	<0.001	Fixed	Overall (7)	0.085	0.946	0.626	0.999	0.131	0.993	0.831	1.000
Pu et al., 2013 [22]	MTHFR C677T	TT+CT vs. CC	1.56 (1.12, 2.18)	0.009	Random	Overall (8)	0.062	0.409	0.993	1.000	0.957	1.000	0.995	1.000
Pu et al., 2013 [22]	MTHFR A1298C	CC vs. AA+AC	0.73 (0.56, 0.97)	0.03	Fixed	Overall (5)	0.181	0.734	0.994	1.000	0.976	1.000	0.997	1.000
Warrier et al., 2015 [24]	SLC25A12/rs2292813	C vs. T	1.372 (1.161, 1.621)	1.97 × 10^−4^	Fixed	Overall (6)	0.058	0.853	0.777	1.000	0.191	0.996	0.877	1.000
Warrier et al., 2015 [24]	CNTNAP2/rs7794745	A vs. T	0.887 (0.828, 0.950)	1.00 × 10^−3^	Fixed	Overall (3)	0.963	1.000	0.389	0.998	0.380	0.998	0.952	1.000
Warrier et al., 2015 [24]	SLC25A12/rs2056202	T vs. C	1.227 (1.079, 1.396)	2.00 × 10^−3^	Fixed	Overall (8)	0.368	0.999	0.837	1.000	0.654	0.999	0.976	1.000
Warrier et al., 2015 [24]	OXTR/rs2268491	T vs. C	1.31 (1.092, 1.572)	4.00 × 10^−3^	Fixed	Overall (2)	0.173	0.927	0.955	1.000	0.799	1.000	0.987	1.000
Warrier et al., 2015 [24]	EN2/rs1861972	A vs. G	1.125 (1.035, 1.224)	6.00 × 10^−3^	Fixed	Overall (8)	0.933	1.000	0.869	1.000	0.861	1.000	0.993	1.000
Warrier et al., 2015 [24]	MTHFR/rs1801133	T vs. C	1.370 (1.079, 1.739)	1.00 × 10^−2^	Random	Overall (10)	0.138	0.772	0.986	1.000	0.926	1.000	0.994	1.000
Warrier et al., 2015 [24]	ASMT/rs4446909	G vs. A	1.195 (1.038, 1.375)	1.30 × 10^−2^	Fixed	Overall (3)	0.523	0.999	0.961	1.000	0.928	1.000	0.995	1.000
Warrier et al., 2015 [24]	MET/rs38845	A vs. G	1.322 (1.013, 1.724)	1.60 × 10^−2^	Random	Overall (3)	0.237	0.824	0.994	1.000	0.979	1.000	0.998	1.000
Warrier et al., 2015 [24]	SLC6A4/rs2020936	T vs. C	1.244 (1.036, 1.492)	1.90 × 10^−2^	Fixed	Overall (4)	0.349	0.978	0.982	1.000	0.950	1.000	0.996	1.000
Warrier et al., 2015 [24]	SLC6A4/STin2 VNTR	12 vs. 9/10	1.492 (1.068, 2.083)	1.90 × 10^−2^	Fixed	Caucasian (4)	0.100	0.513	0.995	1.000	0.973	1.000	0.997	1.000
Warrier et al., 2015 [24]	STX1A/rs4717806	A vs. T	0.851 (0.741, 0.978)	2.30 × 10^−2^	Fixed	Overall (4)	0.616	1.000	0.974	1.000	0.958	1.000	0.997	1.000
Warrier et al., 2015 [24]	RELN/rs736707	T vs. C	1.269 (1.030, 1.563)	2.50 × 10^−2^	Random	Overall (7)	0.299	0.942	0.988	1.000	0.964	1.000	0.997	1.000
Warrier et al., 2015 [24]	PON1/rs662	A vs. G	0.794 (0.642, 0.983)	3.40 × 10^−2^	Fixed	Overall (2)	0.329	0.946	0.990	1.000	0.973	1.000	0.997	1.000
Warrier et al., 2015 [24]	OXTR/rs237887	G vs. A	1.163 (1.002, 1.349)	4.70 × 10^−2^	Fixed	Overall (2)	0.660	1.000	0.986	1.000	0.979	1.000	0.998	1.000
Warrier et al., 2015 [24]	EN2/rs1861973	T vs. C	0.86 (0.791, 0.954)	3.00 × 10^−3^	Fixed	TDT (3)	0.724	1.000	0.858	1.000	0.814	1.000	0.989	1.000
Aoki et al., 2016 [25]	SCL25A12/rs2292813	G (risk allele)	1.190 (1.052, 1.346)	0.006	Random	Overall (9)	0.553	1.000	0.911	1.000	0.849	1.000	0.990	1.000
Aoki et al., 2016 [25]	SCL25A12/rs2056202	G (risk allele)	1.206 (1.035, 1.405)	0.016	Random	Overall (10)	0.474	0.997	0.972	1.000	0.942	1.000	0.996	1.000
LoParo et al., 2015 [26]	OXTR/rs237887	G (minor allele)	0.89 (0.79, 0.98)	0.0239	Random	Overall (3)	0.910	1.000	0.951	1.000	0.947	1.000	0.997	1.000
LoParo et al., 2015 [26]	OXTR/rs2268491	T (minor allele)	1.20 (1.05, 1.35)	0.0075	Random	Overall (3)	0.500	1.000	0.828	1.000	0.707	1.000	0.981	1.000
Wang et al., 2014 [27]	RELN/rs362691	R vs. NR	0.69 (0.56, 0.86)	0.001	Fixed	Overall (7)	0.047	0.620	0.954	1.000	0.607	0.999	0.969	1.000
Torrico et al., 2015 [28]	PTCHD1/rs7052177	T (major allele)	0.58 (0.45, 0.76)	6.8 × 10^−5^	Fixed	European (4) ^†^	0.004	0.156	0.948	1.000	0.333	0.998	0.890	1.000
Kranz et al., 2016 [29]	OXTR/rs237889	A vs. G	1.12 (1.01, 1.24)	0.0365	Random	Overall (3)	0.908	1.000	0.970	1.000	0.967	1.000	0.998	1.000

Abbreviations: A, Adenine; C, Cytosine; G, Guanine; T, Thymine; R, Risk allele; NR, Non-risk allele; FPRP, false positive rate probability; BFDP, Bayesian false discovery probability; OR, odds ratio; CI, confidence interval; NA, not available; The bold in the table means significant results by FPRP and BFDP. ^†^ This article reported only the number of datasets not the number of individual studies included in the meta-analysis. Thus, we wrote the number of datasets in the parenthesis.

**Table 2:** Re-analysis results of gene variants with genome wide statistical significance (*p*-value < 5 × 10^−8^) and borderline statistical significance (5 × 10^−8^ ≤ *p*-value < 0.05) in GWAS meta-analyses.

Author, Year	Gene	Variant	Comparison	OR (95% CI)	*p*-Value	Power OR 1.2	Power OR 1.5	FPRP Values at Prior Probability				BFDP 0.001	BFDP 0.000001
								OR 1.2		OR 1.5			
								0.001	0.000001	0.001	0.000001		
*Gene variants with statistically significance (p-value < 5 × 10^−8^), FPRP < 0.2 and BFDP < 0.8 from meta-analysis of GWAS*													
Anney et al., 2010 [30]	MACROD2	rs4141463	A (minor allele)	0.73 (0.66–0.82)	3.7 × 10^−8^	0.013	0.937	0.009	0.898	0.000	0.107	0.008	0.891
*Gene variants with statistically borderline significance (5 × 10^−8^ ≤ p-value < 0.05), FPRP < 0.2 and BFDP < 0.8 from meta-analyses of GWAS*													
Anney et al., 2017 [31]	ALPK3 NMB SCAND2P SEC11A SLC28A1 WDR73 ZNF592	rs4842996	T vs. C	1.08 (1.05–1.12)	0.00001044	1.000	1.000	0.032	0.971	0.032	0.971	0.688	1.000
	EXOC4	rs6467494	T vs. C	1.07 (1.04–1.09)	0.0000172	1.000	1.000	0.000	0.000	0.000	0.000	0.000	0.000
	NA	rs13233145	A vs. C	1.07 (1.04–1.10)	0.00002906	1.000	1.000	0.002	0.618	0.002	0.618	0.136	0.994
	NA	rs7684366	T vs. C	0.93 (0.90–0.96)	0.00003137	1.000	1.000	0.007	0.882	0.007	0.882	0.373	0.998
	MEGF10	rs73785549	C vs. G	1.15 (1.08–1.21)	0.0001308	0.950	1.000	0.000	0.070	0.000	0.067	0.005	0.835
	ANO4	rs2055471	A vs. T	1.07 (1.03–1.10)	0.0001334	1.000	1.000	0.002	0.618	0.002	0.618	0.136	0.994
	BNC2	rs7860276	A vs. G	1.10 (1.05–1.15)	0.0003196	1.000	1.000	0.026	0.964	0.026	0.964	0.598	0.999
	NA	rs2293280	C vs. G	1.12 (1.06–1.18)	0.0003606	0.995	1.000	0.020	0.954	0.020	0.954	0.514	0.999
	NA	rs16975940	T vs. C	1.07 (1.03–1.10)	0.0004742	1.000	1.000	0.002	0.618	0.002	0.618	0.136	0.994
	NA	rs10169115	C vs. G	1.06 (1.02–1.09)	0.004465	1.000	1.000	0.041	0.977	0.041	0.977	0.778	1.000
	C10orf76 CUEDC2 ELOVL3 FBXL15 GBF1 HPS6 LDB1 MIR146B NFKB2 NOLC1 PITX3 PPRC1 PSD	rs1409313	T vs. C	1.10 (1.06–1.14)	1.467 × 10^−6^	1.000	1.000	0.000	0.145	0.000	0.145	0.014	0.936
	ESRRG	rs12725407	C vs. G	1.10 (1.06–1.14)	2.115 × 10^−6^	1.000	1.000	0.000	0.145	0.000	0.145	0.014	0.936
	HDAC4 MIR2467 MIR4269	rs2931203	A vs. T	0.92 (0.88–0.95)	4.243 × 10^−6^	1.000	1.000	0.000	0.261	0.000	0.261	0.031	0.970
Ma et al., 2009 [32]	NA	rs7704909	C(minor)/T(major)	1.30 (1.15–1.46)	1.53 × 10^−5^	0.088	0.992	0.096	0.991	0.009	0.905	0.295	0.998
	NA	rs1896731	C(minor)/T(major)	0.76 (0.67–0.85)	1.90 × 10^−5^	0.053	0.989	0.028	0.966	0.002	0.609	0.076	0.988
	NA	rs12518194	G(minor)/A(major)	1.31 (1.16–1.49)	8.34 × 10^−6^	0.091	0.980	0.302	0.998	0.039	0.976	0.605	0.999
	NA	rs4307059	C(minor)/T(major)	1.31 (1.16–1.48)	1.29 × 10^−5^	0.079	0.985	0.153	0.995	0.014	0.936	0.383	0.998
	NA	rs4327572	T(minor)/C(major)	1.32 (1.17–1.49)	4.05 × 10^−6^	0.062	0.981	0.103	0.991	0.007	0.878	0.249	0.997
Anney et al., 2010 [30]	NA	rs4078417	C (minor allele)	1.19 (1.10–1.30)	5.6 × 10^−5^	0.574	1.000	0.167	0.995	0.103	0.991	0.795	1.000
	PPP2R5C	rs7142002	G (minor allele)	0.64 (0.53–0.78)	2.9 × 10^−6^	0.004	0.343	0.687	1.000	0.028	0.966	0.459	0.999
Kuo et al., 2015 [33]	NAALADL2	rs3914502	A (minor allele)	1.4 (1.2–1.6)	3.5 × 10^−6^	0.012	0.844	0.062	0.985	0.001	0.482	0.051	0.982
	NAALADL2	rs2222447	A (minor allele)	0.7 (0.6–0.8)	5.3 × 10^−5^	0.005	0.763	0.030	0.969	0.000	0.178	0.013	0.932
	NA	rs12543592	G (minor allele)	0.7 (0.6–0.8)	3.2 × 10^−6^	0.005	0.763	0.030	0.969	0.000	0.178	0.013	0.932
	NA	rs7026342	C (minor allele)	1.6 (1.2–2.0)	1.8 × 10^−4^	0.006	0.285	0.864	1.000	0.113	0.992	0.749	1.000
	NA	rs7030851	A (minor allele)	1.6 (1.3–2.0)	1.4 × 10^−4^	0.006	0.285	0.864	1.000	0.113	0.992	0.749	1.000
Anney et al., 2012 [34]	RASSF5	rs11118968	A	0.44 (0.32–0.61)	2.452 × 10^−7^	0.000	0.006	0.930	1.000	0.117	0.993	0.504	0.999
	DNER	rs6752370	G	1.62 (1.33–1.96)	8.526 × 10^−7^	0.001	0.214	0.407	0.999	0.003	0.764	0.089	0.990
	YEATS2	rs263035	G	1.39 (1.22–1.57)	2.258 × 10^−7^	0.009	0.890	0.013	0.928	0.000	0.115	0.009	0.898
	None	rs29456	A	1.65 (1.37–1.99)	1.226 × 10^−7^	0.000	0.159	0.272	0.997	0.001	0.504	0.028	0.967
	None	rs1936295	A	1.69 (1.37–2.09)	6.636 × 10^−7^	0.001	0.136	0.620	0.999	0.009	0.905	0.179	0.995
	None	rs4761371	A	0.46 (0.34–0.63)	3.914 × 10^−7^	0.000	0.010	0.924	1.000	0.111	0.992	0.521	0.999
	None	rs288604	G	1.58 (1.32–1.88)	2.975 × 10^−7^	0.001	0.279	0.207	0.996	0.001	0.473	0.032	0.971
	MACROD2	rs6110458	A	1.46 (1.27–1.69)	1.806 × 10^−7^	0.004	0.641	0.084	0.989	0.001	0.383	0.033	0.971
	MACROD2 NCRNA00186	rs14135	G	1.49 (1.28–1.74)	1.778 × 10^−7^	0.003	0.534	0.130	0.993	0.001	0.467	0.042	0.977
	NCRNA00186 MACROD2	rs1475531	C	1.53 (1.30–1.79)	2.011 × 10^−7^	0.001	0.402	0.083	0.989	0.000	0.213	0.013	0.929
	PARD3B	rs4675502	NA	1.28 (1.16–1.41)	4.34 × 10^−7^	0.095	0.999	0.006	0.856	0.001	0.362	0.030	0.969
	NA	rs7711337	NA	0.82 (0.76–0.89)	8.25 × 10^−7^	0.350	1.000	0.006	0.854	0.002	0.672	0.091	0.990
	NA	rs7834018	NA	0.64 (0.53–0.77)	7.54 × 10^−7^	0.003	0.333	0.465	0.999	0.007	0.871	0.186	0.996
	TAF1C	rs4150167	NA	0.51 (0.39–0.66)	2.91 × 10^−7^	0.000	0.021	0.764	1.000	0.015	0.937	0.142	0.994
*Gene variants with statistically borderline significance (5 × 10^−8^ ≤ p-value < 0.05), FPRP > 0.2 or BFDP > 0.2 from meta-analyses of GWAS*													
Waltes et al., 2014 [46]	CYFIP1^c^	rs7170637	G > A	0.85 (0.75, 0.96)	0.007	0.625	1.000	0.934	1.000	0.898	1.000	0.993	1.000
	CAMK4^c^	rs25925	C > G	1.31 (1.04, 1.64)	0.021	0.222	0.881	0.988	1.000	0.954	1.000	0.996	1.000
Anney et al., 2017 [31]	NA	rs1436358	T vs. C	0.86 (0.79–0.93)	0.00001473	0.785	1.000	0.168	0.995	0.137	0.994	0.844	1.000
	MACROD2 MACROD2-AS1	rs6079556	A vs. C	0.94 (0.91–0.97)	0.00001731	1.000	1.000	0.102	0.991	0.102	0.991	0.887	1.000
	LINC00535	chr8_94389815_I	I vs. D	0.92 (0.89–0.96)	0.00002102	1.000	1.000	0.109	0.992	0.109	0.992	0.867	1.000
	LINCR-0001 PRSS55	rs4840484	T vs. C	1.07 (1.04–1.11)	0.00002307	1.000	1.000	0.232	0.997	0.232	0.997	0.945	1.000
Anney et al., 2017 (continued)	ADTRP	rs10947543	C vs. G	0.94 (0.91–0.97)	0.000031	1.000	1.000	0.102	0.991	0.102	0.991	0.887	1.000
	LRRC4 MIR593 SND1 SND1-IT1	chr7_127644308_D	D vs. I	0.93 (0.90–0.97)	0.00003235	1.000	1.000	0.422	0.999	0.422	0.999	0.972	1.000
	CCDC93 DDX18 INSIG2	chr2_118616767_D	I vs. D	0.85 (0.78–0.93)	0.00003531	0.667	1.000	0.374	0.998	0.285	0.997	0.921	1.000
	NA	chr14_99235398_I	I vs. D	0.87 (0.81–0.94)	0.00003765	0.862	1.000	0.327	0.998	0.296	0.998	0.930	1.000
	TTBK1	rs2756174	A vs. C	0.94 (0.91–0.97)	0.00005245	1.000	1.000	0.102	0.991	0.102	0.991	0.887	1.000
	HCG4B HLA-A HLA-H	rs115254791	T vs. G	0.94 (0.90–0.97)	0.00005321	1.000	1.000	0.102	0.991	0.102	0.991	0.887	1.000
	MIR2113	rs9482120	A vs. C	0.94 (0.91–0.97)	0.00009513	1.000	1.000	0.102	0.991	0.102	0.991	0.887	1.000
	CRTAP SUSD5	chr3_33191013_D	I vs. D	0.93 (0.89–0.97)	0.0000957	1.000	1.000	0.422	0.999	0.422	0.999	0.972	1.000
	NA	rs9285005	A vs. G	0.91 (0.86–0.96)	0.0001147	0.999	1.000	0.354	0.998	0.354	0.998	0.956	1.000
	LOC100505609	rs73065342	T vs. C	0.89 (0.83–0.95)	0.0001169	0.976	1.000	0.322	0.998	0.317	0.998	0.941	1.000
	DCAF4 DPF3 PAPLN PSEN1 RBM25 ZFYVE1	rs1203311	A vs. C	0.86 (0.79–0.94)	0.0001394	0.756	1.000	0.540	0.999	0.470	0.999	0.960	1.000
	MACROD2	rs192259652	A vs. T	0.91 (0.85–0.96)	0.0001438	0.999	1.000	0.354	0.998	0.354	0.998	0.956	1.000
	FOXP1	rs76188283	T vs. C	1.09 (1.05–1.14)	0.0002093	1.000	1.000	0.142	0.994	0.142	0.994	0.892	1.000
	CCDC38 NTN4 SNRPF	chr12_96221819_D	I vs. D	0.94 (0.91–0.97)	0.0002128	1.000	1.000	0.102	0.991	0.102	0.991	0.887	1.000
	NA	chr3_182308608_I	D vs. I	0.94 (0.90–0.97)	0.0002755	1.000	1.000	0.102	0.991	0.102	0.991	0.887	1.000
	ASTN2 PAPPA PAPPA-AS1	rs7026354	A vs. G	1.05 (1.03–1.08)	0.0003018	1.000	1.000	0.407	0.999	0.407	0.999	0.979	1.000
	NA	rs2368140	A vs. G	0.94 (0.91–0.98)	0.0003049	1.000	1.000	0.783	1.000	0.783	1.000	0.993	1.000
	NA	rs13016472	T vs. C	0.94 (0.91–0.98)	0.0003629	1.000	1.000	0.783	1.000	0.783	1.000	0.993	1.000
	DSCAM	rs62235658	T vs. C	0.92 (0.87–0.97)	0.0004132	1.000	1.000	0.668	1.000	0.668	1.000	0.986	1.000
	NA	rs3113169	C vs. G	0.93 (0.90–0.97)	0.0004234	1.000	1.000	0.422	0.999	0.422	0.999	0.972	1.000
	CASKIN2 GGA3 GRB2 LOC100287042 MIF4GD MIR3678 MIR6785 MRPS7 NUP85 SLC25A19 TMEM94 TSEN54	rs12950709	A vs. G	0.92 (0.87–0.97)	0.0004387	1.000	1.000	0.668	1.000	0.668	1.000	0.986	1.000
	CAMP CDC25A CSPG5 DHX30 MAP4 MIR1226 MIR4443 SMARCC1 ZNF589	rs7429990	A vs. C	0.94 (0.91–0.97)	0.0004525	1.000	1.000	0.102	0.991	0.102	0.991	0.887	1.000
	NA	chr8_84959513_D	D vs. I	0.89 (0.83–0.96)	0.0004634	0.956	1.000	0.728	1.000	0.718	1.000	0.985	1.000
	ACTN2	rs4659712	A vs. G	0.95 (0.92–0.98)	0.0004976	1.000	1.000	0.550	0.999	0.550	0.999	0.986	1.000
	ASB4	rs113706540	T vs. C	0.93 (0.88–0.97)	0.0005006	1.000	1.000	0.422	0.999	0.422	0.999	0.972	1.000
	GJD4	rs7897060	C vs. G	0.95 (0.91–0.98)	0.0005789	1.000	1.000	0.550	0.999	0.550	0.999	0.986	1.000
	AK5 DNAJB4 FAM73A FUBP1 GIPC2 MGC27382 NEXN NEXN-AS1 USP33 ZZZ3	rs12126604	T vs. C	0.92 (0.87–0.97)	0.0006161	1.000	1.000	0.668	1.000	0.668	1.000	0.986	1.000
	SEMA6D	rs17387110	T vs. G	0.95 (0.92–0.98)	0.0006996	1.000	1.000	0.550	0.999	0.550	0.999	0.986	1.000
	NA	chr16_62649826_D	D vs. I	0.87 (0.80–0.95)	0.0007369	0.831	1.000	0.697	1.000	0.657	0.999	0.979	1.000
	NA	rs4239875	A vs. G	1.06 (1.03–1.10)	0.0008018	1.000	1.000	0.672	1.000	0.672	1.000	0.990	1.000
	CTNNA3 DNAJC12 HERC4 MYPN POU5F1P5 SIRT1	chr10_69763783_D	I vs. D	0.91 (0.86–0.97)	0.0008401	0.997	1.000	0.792	1.000	0.791	1.000	0.991	1.000
	CLIC5 ENPP4 ENPP5	rs7762549	A vs. G	0.95 (0.92–0.98)	0.00085	1.000	1.000	0.550	0.999	0.550	0.999	0.986	1.000
	NA	chr18_76035713_D	D vs. I	0.93 (0.88–0.97)	0.000884	1.000	1.000	0.422	0.999	0.422	0.999	0.972	1.000
	BRICD5 CASKIN1 DNASE1L2 E4F1 MIR3180-5 MIR4516 MLST8 PGP PKD1 RAB26 SNHG19 SNORD60 TRAF7	rs2078282	A vs. G	0.94 (0.91–0.98)	0.0009187	1.000	1.000	0.783	1.000	0.783	1.000	0.993	1.000
	OPCML	rs7952100	C vs. G	1.06 (1.03–1.10)	0.0009399	1.000	1.000	0.672	1.000	0.672	1.000	0.990	1.000
	LOC101927907 LRRTM4	rs58500924	A vs. G	0.90 (0.84–0.96)	0.0009721	0.990	1.000	0.581	0.999	0.579	0.999	0.977	1.000
	RNGTT	rs35675874	A vs. G	0.94 (0.91–0.98)	0.001031	1.000	1.000	0.783	1.000	0.783	1.000	0.993	1.000
	LOC101928505 LOC101928539	chr5_57079215_I	D vs. I	1.07 (1.03–1.11)	0.001076	1.000	1.000	0.232	0.997	0.232	0.997	0.945	1.000
	DPP4 SLC4A10	rs2909451	T vs. C	0.94 (0.90–0.98)	0.001078	1.000	1.000	0.783	1.000	0.783	1.000	0.993	1.000
	ERAP2 LNPEP	rs55767008	T vs. C	0.89 (0.82–0.96)	0.001182	0.956	1.000	0.728	1.000	0.718	1.000	0.985	1.000
	C2orf15 KIAA1211L LIPT1 LOC101927070 TSGA10	rs10202643	A vs. T	0.95 (0.92–0.98)	0.001269	1.000	1.000	0.550	0.999	0.550	0.999	0.986	1.000
	AUTS2	rs2293507	T vs. G	0.88 (0.81–0.96)	0.001337	0.890	1.000	0.817	1.000	0.799	1.000	0.989	1.000
	NA	rs138457704	A vs. G	1.07 (1.03–1.11)	0.001357	1.000	1.000	0.232	0.997	0.232	0.997	0.945	1.000
	GLDC	rs13288399	C vs. G	0.95 (0.91–0.98)	0.001357	1.000	1.000	0.550	0.999	0.550	0.999	0.986	1.000
	MTFR1 PDE7A	rs1513723	C vs. G	0.95 (0.92–0.98)	0.001447	1.000	1.000	0.550	0.999	0.550	0.999	0.986	1.000
	ASTN2 ASTN2-AS1 PAPPA TRIM32	rs146737360	T vs. G	0.95 (0.92–0.98)	0.001534	1.000	1.000	0.550	0.999	0.550	0.999	0.986	1.000
	NA	chr6_45726254_D	D vs. I	0.90 (0.83–0.96)	0.001606	0.990	1.000	0.581	0.999	0.579	0.999	0.977	1.000
	NA	rs6742513	C vs. G	1.07 (1.03–1.11)	0.001611	1.000	1.000	0.232	0.997	0.232	0.997	0.945	1.000
	NA	rs73204738	A vs. C	0.92 (0.88–0.97)	0.001617	1.000	1.000	0.668	1.000	0.668	1.000	0.986	1.000
	LINC01553	rs11817353	A vs. C	0.95 (0.92–0.98)	0.001678	1.000	1.000	0.550	0.999	0.550	0.999	0.986	1.000
Anney et al., 2017 (continued)	RAD51B	rs2842330	A vs. C	1.10 (1.04–1.16)	0.001845	0.999	1.000	0.303	0.998	0.303	0.998	0.946	1.000
	RBFOX1	rs12930616	C vs. G	1.05 (1.02–1.09)	0.001985	1.000	1.000	0.913	1.000	0.913	1.000	0.998	1.000
	GRID2	rs6811974	T vs. C	0.95 (0.93–0.98)	0.001995	1.000	1.000	0.550	0.999	0.550	0.999	0.986	1.000
	NA	rs7135621	T vs. C	0.96 (0.93–0.98)	0.002059	1.000	1.000	0.094	0.991	0.094	0.991	0.915	1.000
	GFER NOXO1 NPW RNF151 RPS2 SNHG9 SNORA78 SYNGR3 TBL3 ZNF598	rs55742253	T vs. C	0.93 (0.88–0.98)	0.002075	1.000	1.000	0.868	1.000	0.868	1.000	0.995	1.000
	PTPRB	rs10784860	T vs. C	0.95 (0.91–0.98)	0.002211	1.000	1.000	0.550	0.999	0.550	0.999	0.986	1.000
	LOC101927768	rs9387201	C vs. G	1.09 (1.03–1.14)	0.002427	1.000	1.000	0.142	0.994	0.142	0.994	0.892	1.000
	BTBD11 LOC101929162 PRDM4 PWP1	rs4964602	T vs. G	0.95 (0.91–0.98)	0.00256	1.000	1.000	0.550	0.999	0.550	0.999	0.986	1.000
	NA	rs1376888	T vs. C	1.05 (1.02–1.08)	0.002668	1.000	1.000	0.407	0.999	0.407	0.999	0.979	1.000
	KLHL29	rs10182178	A vs. G	1.05 (1.02–1.08)	0.003508	1.000	1.000	0.407	0.999	0.407	0.999	0.979	1.000
	UBE2H	rs78661858	A vs. G	0.91 (0.85–0.97)	0.003665	0.997	1.000	0.792	1.000	0.791	1.000	0.991	1.000
	VAPA	rs29063	A vs. G	1.04 (1.01–1.07)	0.004075	1.000	1.000	0.873	1.000	0.873	1.000	0.997	1.000
	NA	rs190401890	A vs. T	1.12 (1.04–1.20)	0.004114	0.975	1.000	0.568	0.999	0.562	0.999	0.975	1.000
	LOC102723427	rs192668887	T vs. C	0.91 (0.84–0.97)	0.004205	0.997	1.000	0.792	1.000	0.791	1.000	0.991	1.000
	SLC12A7	rs73031119	A vs. C	0.91 (0.84–0.97)	0.004399	0.997	1.000	0.792	1.000	0.791	1.000	0.991	1.000
	ADGRL2	rs75695875	A vs. G	0.93 (0.87–0.98)	0.004715	1.000	1.000	0.868	1.000	0.868	1.000	0.995	1.000
	NA	rs1943999	C vs. G	0.96 (0.92–0.99)	0.004915	1.000	1.000	0.903	1.000	0.903	1.000	0.998	1.000
	DNAH6	rs2222734	A vs. G	0.92 (0.87–0.98)	0.005058	0.999	1.000	0.906	1.000	0.906	1.000	0.996	1.000
	OR8A1 OR8B12	rs2226753	T vs. C	0.96 (0.93–0.99)	0.005074	1.000	1.000	0.903	1.000	0.903	1.000	0.998	1.000
	TUSC5	rs35713482	A vs. G	1.05 (1.01–1.08)	0.005154	1.000	1.000	0.407	0.999	0.407	0.999	0.979	1.000
	C5orf15 VDAC1	rs67120295	T vs. C	1.06 (1.02–1.10)	0.005745	1.000	1.000	0.672	1.000	0.672	1.000	0.990	1.000
	NA	rs76010911	A vs. G	1.11 (1.04–1.19)	0.006255	0.986	1.000	0.769	1.000	0.767	1.000	0.989	1.000
	MTMR9 SLC35G5 TDH	rs6601581	T vs. C	1.06 (1.02–1.11)	0.006463	1.000	1.000	0.930	1.000	0.930	1.000	0.998	1.000
	HSDL2 MIR3134 PTBP3 SUSD1	rs7024761	A vs. G	1.05 (1.02–1.09)	0.00648	1.000	1.000	0.913	1.000	0.913	1.000	0.998	1.000
	CRTC3 GABARAPL3 IQGAP1 ZNF774	rs2601187	A vs. G	1.05 (1.01–1.08)	0.006859	1.000	1.000	0.407	0.999	0.407	0.999	0.979	1.000
	LOC101927189 LRRC1	rs4715431	A vs. G	1.04 (1.01–1.08)	0.007007	1.000	1.000	0.977	1.000	0.977	1.000	0.999	1.000
	NA	rs646680	A vs. G	0.95 (0.92–0.99)	0.00723	1.000	1.000	0.937	1.000	0.937	1.000	0.998	1.000
	CCNE1	rs12609867	A vs. G	0.95 (0.91–0.99)	0.00743	1.000	1.000	0.937	1.000	0.937	1.000	0.998	1.000
	NOS1AP OLFML2B	rs75192393	T vs. C	1.07 (1.02–1.12)	0.007697	1.000	1.000	0.787	1.000	0.787	1.000	0.993	1.000
	KDM4A KDM4A-AS1 LOC101929592 MIR6079 PTPRF ST3GAL3	rs79857083	T vs. C	1.04 (1.01–1.08)	0.007758	1.000	1.000	0.977	1.000	0.977	1.000	0.999	1.000
	NA	rs142968358	T vs. G	1.04 (1.01–1.07)	0.007789	1.000	1.000	0.873	1.000	0.873	1.000	0.997	1.000
	C3orf30 IGSF11 IGSF11-AS1 UPK1B	rs1102586	A vs. G	1.06 (1.02–1.10)	0.007844	1.000	1.000	0.672	1.000	0.672	1.000	0.990	1.000
	NA	chr11_98107192_D	D vs. I	1.04 (1.01–1.08)	0.00785	1.000	1.000	0.977	1.000	0.977	1.000	0.999	1.000
	C9orf135	rs76014157	A vs. G	0.90 (0.82–0.98)	0.007946	0.962	1.000	0.941	1.000	0.939	1.000	0.997	1.000
	NA	rs6437449	A vs. G	1.07 (1.02–1.11)	0.008708	1.000	1.000	0.232	0.997	0.232	0.997	0.945	1.000
	MYO5A	chr15_52811815_D	I vs. D	0.90 (0.81–0.98)	0.008799	0.962	1.000	0.941	1.000	0.939	1.000	0.997	1.000
	NA	rs9466619	A vs. G	0.95 (0.92–0.99)	0.009071	1.000	1.000	0.937	1.000	0.937	1.000	0.998	1.000
	NA	rs6117854	A vs. G	0.96 (0.93–0.99)	0.01012	1.000	1.000	0.903	1.000	0.903	1.000	0.998	1.000
	C7orf33	rs6955951	A vs. T	1.04 (1.01–1.07)	0.01015	1.000	1.000	0.873	1.000	0.873	1.000	0.997	1.000
	LHX6	rs72767788	A vs. C	0.95 (0.91–0.99)	0.01093	1.000	1.000	0.937	1.000	0.937	1.000	0.998	1.000
	NA	rs2028664	A vs. C	1.04 (1.01–1.07)	0.01095	1.000	1.000	0.873	1.000	0.873	1.000	0.997	1.000
	ELAVL2	rs180861134	A vs. T	1.05 (1.01–1.09)	0.01104	1.000	1.000	0.913	1.000	0.913	1.000	0.998	1.000
	RASGEF1C	rs12659560	T vs. C	1.04 (1.01–1.07)	0.0112	1.000	1.000	0.873	1.000	0.873	1.000	0.997	1.000
	MIR548AZ SYNE2	rs2150291	T vs. C	1.05 (1.01–1.09)	0.0113	1.000	1.000	0.913	1.000	0.913	1.000	0.998	1.000
	WDFY4	rs118059975	A vs. C	0.95 (0.91–0.99)	0.01146	1.000	1.000	0.937	1.000	0.937	1.000	0.998	1.000
	LINC01525 MAN1A2	rs3820500	A vs. G	1.04 (1.01–1.07)	0.0116	1.000	1.000	0.873	1.000	0.873	1.000	0.997	1.000
	GALNT10	rs17629195	T vs. C	1.04 (1.01–1.07)	0.012	1.000	1.000	0.873	1.000	0.873	1.000	0.997	1.000
	MIR597 TNKS	rs78853604	T vs. C	1.05 (1.01–1.08)	0.01256	1.000	1.000	0.407	0.999	0.407	0.999	0.979	1.000
	EXT1	rs7835763	A vs. T	1.04 (1.01–1.08)	0.01283	1.000	1.000	0.977	1.000	0.977	1.000	0.999	1.000
	NA	rs4652928	A vs. G	0.96 (0.92–0.99)	0.01384	1.000	1.000	0.903	1.000	0.903	1.000	0.998	1.000
	PDE1C	rs11976985	T vs. C	0.95 (0.92–0.99)	0.0141	1.000	1.000	0.937	1.000	0.937	1.000	0.998	1.000
	BAX FTL GYS1	rs2230267	T vs. C	1.04 (1.01–1.07)	0.01429	1.000	1.000	0.873	1.000	0.873	1.000	0.997	1.000
Anney et al., 2017 (continued)	GRID2	rs6854329	C vs. G	0.92 (0.86–0.99)	0.01486	0.996	1.000	0.963	1.000	0.963	1.000	0.998	1.000
	NA	rs1926229	C vs. G	1.05 (1.01–1.08)	0.01496	1.000	1.000	0.407	0.999	0.407	0.999	0.979	1.000
	NA	rs261351	T vs. C	0.96 (0.93–0.99)	0.01498	1.000	1.000	0.903	1.000	0.903	1.000	0.998	1.000
	RAPGEF2	rs4440173	A vs. G	1.04 (1.01–1.07)	0.01564	1.000	1.000	0.873	1.000	0.873	1.000	0.997	1.000
	MIR4650-1 MIR4650-2 POM121 SBDSP1 SPDYE7P TYW1B	rs4392770	T vs. C	1.05 (1.01–1.09)	0.01564	1.000	1.000	0.913	1.000	0.913	1.000	0.998	1.000
	NA	rs138493916	C vs. G	1.08 (1.02–1.14)	0.01783	1.000	1.000	0.840	1.000	0.840	1.000	0.994	1.000
	NA	rs615512	A vs. G	1.08 (1.02–1.14)	0.01811	1.000	1.000	0.840	1.000	0.840	1.000	0.994	1.000
	EP400 EP400NL PUS1 SNORA49	rs11608890	T vs. G	0.94 (0.88–0.99)	0.0187	1.000	1.000	0.951	1.000	0.951	1.000	0.998	1.000
	DIAPH3	chr13_60161890_I	I vs. D	1.05 (1.01–1.09)	0.01984	1.000	1.000	0.913	1.000	0.913	1.000	0.998	1.000
	ADAM12	rs1674923	T vs. C	0.96 (0.93–0.99)	0.0203	1.000	1.000	0.903	1.000	0.903	1.000	0.998	1.000
	ATP2B2 GHRL GHRLOS IRAK2 LINC00852 MIR378B MIR885 SEC13 TATDN2	rs7619385	A vs. G	1.04 (1.01–1.07)	0.02102	1.000	1.000	0.873	1.000	0.873	1.000	0.997	1.000
	UNC13C	rs75099274	A vs. G	1.08 (1.01–1.14)	0.02123	1.000	1.000	0.840	1.000	0.840	1.000	0.994	1.000
	ZSWIM6	rs10053166	A vs. G	0.95 (0.90–0.99)	0.02226	1.000	1.000	0.937	1.000	0.937	1.000	0.998	1.000
	HIVEP3	rs2786484	T vs. C	0.93 (0.86–0.99)	0.0237	1.000	1.000	0.958	1.000	0.958	1.000	0.998	1.000
	FJX1 TRIM44	rs76847144	T vs. C	0.93 (0.86–0.99)	0.02643	1.000	1.000	0.958	1.000	0.958	1.000	0.998	1.000
	WBSCR17	rs148521358	C vs. G	0.94 (0.88–0.99)	0.02731	1.000	1.000	0.951	1.000	0.951	1.000	0.998	1.000
	MIR3134 SUSD1	rs2564899	T vs. C	0.97 (0.94–1.00)	0.02735	1.000	1.000	0.980	1.000	0.980	1.000	0.999	1.000
	NA	chr8_138837351_I	I vs. D	1.05 (1.01–1.09)	0.0284	1.000	1.000	0.913	1.000	0.913	1.000	0.998	1.000
	LINC01393 MDFIC	rs7799732	A vs. G	1.03 (1.00–1.06)	0.03114	1.000	1.000	0.978	1.000	0.978	1.000	0.999	1.000
	TBX18 TBX18-AS1	rs76397051	A vs. G	1.05 (1.01–1.10)	0.034	1.000	1.000	0.975	1.000	0.975	1.000	0.999	1.000
	NA	rs171794	T vs. C	1.06 (1.01–1.12)	0.03587	1.000	1.000	0.974	1.000	0.974	1.000	0.999	1.000
	GDA	rs4327921	A vs. G	0.97 (0.94–1.00)	0.03938	1.000	1.000	0.980	1.000	0.980	1.000	0.999	1.000
	NA	rs2167341	T vs. G	1.05 (1.00–1.10)	0.04203	1.000	1.000	0.975	1.000	0.975	1.000	0.999	1.000
	EVA1C	rs62216215	A vs. C	1.04 (1.00–1.08)	0.04598	1.000	1.000	0.977	1.000	0.977	1.000	0.999	1.000
	LINC01036	rs17589281	T vs. C	0.95 (0.89–1.00)	0.04716	1.000	1.000	0.980	1.000	0.980	1.000	0.999	1.000
	LOC283585	rs61979775	T vs. C	0.97 (0.93–1.00)	0.04813	1.000	1.000	0.980	1.000	0.980	1.000	0.999	1.000
	CHMP4A GMPR2 MDP1 NEDD8 NEDD8-MDP1 TM9SF1 TSSK4	rs72694312	T vs. G	1.06 (1.00–1.11)	0.04814	1.000	1.000	0.930	1.000	0.930	1.000	0.998	1.000
Ma et al., 2009 [32]	NA	rs10065041	T(minor)/C(major)	1.21 (1.08–1.36)	3.24 × 10^−4^	0.445	1.000	0.757	1.000	0.581	0.999	0.970	1.000
	NA	rs10038113	C(minor)/T(major)	0.75 (0.70–0.90)	3.40 × 10^−6^	0.129	0.897	0.939	1.000	0.688	1.000	0.979	1.000
	NA	rs6894838	T(minor)/C(major)	1.26 (1.12–1.42)	8.00 × 10^−5^	0.212	0.998	0.416	0.999	0.131	0.993	0.827	1.000
Anney et al., 2010 [30]	HAT1	rs6731562	G (minor allele)	1.25 (1.11–1.41)	2.0 × 10^−4^	0.253	0.998	0.527	0.999	0.220	0.996	0.891	1.000
	POU6F2	rs10258862	G (minor allele)	1.09 (1.00–1.18)	4.6 × 10^−2^	0.991	1.000	0.971	1.000	0.971	1.000	0.998	1.000
	NA	rs6557675	A (minor allele)	0.84 (0.76–0.93)	1.0 × 10^−3^	0.561	1.000	0.583	0.999	0.440	0.999	0.953	1.000
	MYH11	rs17284809	A (minor allele)	0.63 (0.50–0.79)	5.7 × 10^−5^	0.008	0.312	0.891	1.000	0.168	0.995	0.821	1.000
	GSG1L	rs205409	G (minor allele)	0.91 (0.84–0.99)	2.8 × 10^−2^	0.980	1.000	0.966	1.000	0.966	1.000	0.998	1.000
	TAF1C	rs4150167	A (minor allele)	0.54 (0.40–0.73)	2.1 × 10^−5^	0.002	0.085	0.963	1.000	0.420	0.999	0.905	1.000
Kuo et al., 2015 [33]	GLIS1	rs12082358	C (minor allele)	1.3 (1.1–1.5)	2.2 × 10^−4^	0.136	0.975	0.705	1.000	0.251	0.997	0.906	1.000
	GLIS1	rs12080993	A (minor allele)	1.3 (1.1–1.5)	1.5 × 10^−4^	0.136	0.975	0.705	1.000	0.251	0.997	0.906	1.000
	GPD2	rs3916984	A (minor allele)	1.3 (1.1–1.5)	3.1 × 10^−4^	0.136	0.975	0.705	1.000	0.251	0.997	0.906	1.000
	LRP2/BBS5	rs13014164	C (minor allele)	1.7 (1.3–2.3)	8.6 × 10^−5^	0.012	0.209	0.980	1.000	0.735	1.000	0.974	1.000
	PDGFRA	rs7697680	G (minor allele)	1.5 (1.2–1.9)	9.2 × 10^−4^	0.032	0.500	0.960	1.000	0.607	0.999	0.967	1.000
	FSTL4	rs11741756	A (minor allele)	1.3 (1.1–1.5)	1.2 × 10^−2^	0.136	0.975	0.705	1.000	0.251	0.997	0.906	1.000
	NA	rs13211684	G (minor allele)	1.3 (1.1–1.5)	2.5 × 10^−3^	0.136	0.975	0.705	1.000	0.251	0.997	0.906	1.000
	NA	rs10966205	T (minor allele)	1.3 (1.2–1.5)	2.9 × 10^−5^	0.136	0.975	0.705	1.000	0.251	0.997	0.906	1.000
	C10orf68	rs10763893	A (minor allele)	1.6 (1.2–2.2)	6.1 × 10^−4^	0.038	0.346	0.990	1.000	0.917	1.000	0.992	1.000
	NA	rs12366025	A (minor allele)	1.3 (1.1–1.6)	3.8 × 10^−3^	0.225	0.912	0.983	1.000	0.936	1.000	0.995	1.000
	NA	rs11030597	G (minor allele)	1.3 (1.1–1.6)	4.1 × 10^−3^	0.225	0.912	0.983	1.000	0.936	1.000	0.995	1.000
	NA	rs7933990	A (minor allele)	1.3 (1.1–1.6)	2.5 × 10^−3^	0.225	0.912	0.983	1.000	0.936	1.000	0.995	1.000
	NA	rs11030606	A (minor allele)	1.3 (1.1–1.6)	5.6 × 10^−3^	0.225	0.912	0.983	1.000	0.936	1.000	0.995	1.000
	MACROD2	rs17263514	A (minor allele)	1.2 (1.0–1.4)	1.4 × 10^−2^	0.500	0.998	0.976	1.000	0.953	1.000	0.996	1.000
	BCAS1/CYP24A1	rs12479663	C (minor allele)	1.5 (1.3–1.9)	4.0 × 10^−5^	0.032	0.500	0.960	1.000	0.607	0.999	0.967	1.000

Abbreviations: A, Adenine; C, Cytosine; G, Guanine; T, Thymine; D, Deletion; I, Insertion; R, Risk allele; NR, Non-risk allele; FPRP, false positive rate probability; BFDP, Bayesian false discovery probability; OR, odds ratio; CI, confidence interval; NA, not available.

**Table 3:** Re-analysis results of gene variants with genome wide statistical significance (*p*-value < 5 × 10^−8^) and borderline statistical significance (5 × 10^−8^ ≤ *p*-value < 0.05) in the genome-wide association studies (GWAS) catalog.

Author, Year	Gene	Variant	Comparison	OR (95% CI)	*p*-Value	Power OR 1.2	Power OR 1.5	FPRP Values at Prior Probability				BFDP 0.001	BFDP 0.000001
								OR 1.2		OR 1.5			
								0.001	0.000001	0.001	0.000001		
*Gene variants with statistically significance (p-value < 5 × 10^−8^), FPRP < 0.2 and BFDP < 0.8 from GWAS catalog*													
Anney et al., 2010 [30]	MACROD2	rs4141463	NA	1.37 (1.22–1.52)	4.00 × 10^−8^	0.006	0.956	0.000	0.316	0.000	0.003	0.000	0.208
Chaste et al., 2014 [35]	AL163541.1	rs4773054	NA	2.66 (1.83–3.86)	5.00 × 10^−8^	0.000	0.001	0.949	1.000	0.169	0.995	0.526	0.999
*Gene variants with statistically borderline significance (5 × 10^−8^ ≤ p-value < 0.05), FPRP < 0.2 and BFDP < 0.8 from GWAS catalog*													
Anney et al., 2010 [30]	PPP2R5C	rs7142002	NA	1.56 (1.28–1.89)	3.00 × 10^−6^	0.004	0.344	0.602	0.999	0.016	0.942	0.338	0.998
Anney et al., 2012 [34]	TAF1C	rs4150167	NA	1.96 (1.52–2.56)	3.00 × 10^−7^	0.000	0.025	0.832	1.000	0.031	0.969	0.269	0.997
Anney et al., 2012 [34]	PARD3B	rs4675502	NA	1.28 (1.16–1.41)	4.00 × 10^−7^	0.095	0.999	0.006	0.856	0.001	0.362	0.030	0.969
Anney et al., 2012 [34]	AC113414.1	rs7711337	NA	1.22 (1.12–1.32)	8.00 × 10^−7^	0.340	1.000	0.002	0.689	0.001	0.429	0.038	0.975
Anney et al., 2012 [34]	AC009446.1, EYA1	rs7834018	NA	1.56 (1.3–1.89)	8.00 × 10^−7^	0.004	0.344	0.602	0.999	0.016	0.942	0.338	0.998
Anney et al., 2017 [31]	AL133270.1, AL139093.1	rs142968358	T (risk allele)	1.1 (1.06–1.14)	1.00 × 10^−6^	1.000	1.000	0.000	0.145	0.000	0.145	0.014	0.936
Anney et al., 2017 [31]	EXT1	rs7835763	A (risk allele)	1.1 (1.06–1.14)	2.00 × 10^−6^	1.000	1.000	0.000	0.145	0.000	0.145	0.014	0.936
Chaste et al., 2014 [35]	INHCAP	rs1867503	NA	1.55 (1.30–1.84)	4.00 × 10^−7^	0.002	0.354	0.241	0.997	0.002	0.608	0.058	0.984
Chaste et al., 2014 [35]	CUEDC2	rs1409313	NA	1.75 (1.40–2.18)	4.00 × 10^−7^	0.000	0.085	0.610	0.999	0.007	0.876	0.121	0.993
Chaste et al., 2014 [35]	CTU2	rs11641365	NA	2.06 (1.54–2.76)	3.00 × 10^−7^	0.000	0.017	0.897	1.000	0.071	0.987	0.433	0.999
Chaste et al., 2014 [35]	AC067752.1, AC024598.1, ZNF365	rs93895	NA	1.91 (1.48–2.47)	2.00 × 10^−7^	0.000	0.033	0.804	1.000	0.024	0.961	0.241	0.997
Kuo et al., 2015 [33]	LINC01151, AC108136.1	rs12543592	G (risk allele)	1.43 (1.25–1.67)	3.00 × 10^−6^	0.013	0.727	0.318	0.998	0.008	0.895	0.275	0.997
Kuo et al., 2015 [33]	NAALADL2	rs3914502	A (risk allele)	1.4 (1.20–1.60)	4.00 × 10^−6^	0.012	0.844	0.062	0.985	0.001	0.482	0.051	0.982
Kuo et al., 2015 [33]	OR2M4	rs10888329	NA	1.82 (1.39–2.33)	8.00 × 10^−6^	0.000	0.062	0.809	1.000	0.031	0.970	0.338	0.998
Kuo et al., 2015 [33]	SGSM2	rs2447097	A (risk allele)	1.53 (1.27–1.85)	9.00 × 10^−6^	0.006	0.419	0.652	0.999	0.026	0.965	0.467	0.999
Ma et al., 2009 [32]	Intergenic (RNU6-374P - MSNP1)	rs10038113	T (risk allele)	1.33 (1.11–1.43]	3.00 × 10^−6^	0.003	0.999	0.000	0.000	0.000	0.000	0.000	0.000
*Gene variants with statistically borderline significance (5 × 10^-8^≤ p-value < 0.05), FPRP > 0.2 or BFDP > 0.8 from GWAS catalog*													
Chaste et al., 2014 [35]	AL163541.1	rs4773054	NA	2.9 (1.91–4.39)	7.00 × 10^−8^	0.000	0.001	0.970	1.000	0.345	0.998	0.741	1.000
Anney et al., 2017 [31]	HLA-A, AL671277.1	rs115254791	G (risk allele)	1.0869565 (1.05–1.14)	4.00 × 10^−6^	1.000	1.000	0.376	0.998	0.376	0.998	0.963	1.000

Abbreviations: A, Adenine; G; Guanine; T, Thymine; FPRP, false positive rate probability; BFDP, Bayesian false discovery probability; OR, odds ratio; CI, confidence interval; F, fixed effects model; R, random effects model; NA, not available; ASD, autism spectrum disorder.

**Table 4:** Re-analysis results of gene variants with genome wide statistical significance (*p*-value < 5 × 10^−8^) and borderline statistical significance (5 × 10^−8^ ≤ *p*-value < 0.05) in the GWAS datasets included in GWAS meta-analyses (results of FPRP < 0.2 and BFDP < 0.8).

Author, Year	Trait	Gene(s)	Variant	Comparison	OR (95% CI)	*p*-Value	Power OR 1.2	Power OR 1.5	FPRP Values at Prior Probability				BFDP 0.001	BFDP 0.000001
									OR 1.2		OR 1.5			
									0.001	0.000001	0.001	0.000001		
Anney et al., 2012 [34]	ASD (European)	ERBB4	rs1879532	A	2.02 (1.57–2.59)	1.55 × 10^−8^	0.000	0.009	0.595	0.999	0.003	0.757	0.026	0.964
Anney et al., 2012 [34]	Autism (European)	None	rs289932	A	0.49 (0.38–0.64)	5.04 × 10^−8^	0.000	0.012	0.772	1.000	0.014	0.932	0.114	0.992
Anney et al., 2012 [34]	ASD	TMEM132B	rs16919315	A	0.53 (0.42–0.67)	5.12 × 10^−8^	0.000	0.028	0.589	0.999	0.004	0.800	0.049	0.981
Anney et al., 2012 [34]	Autism (European)	ERBB4	rs1879532	A	1.72 (1.39–2.11)	1.66 × 10^−7^	0.000	0.095	0.416	0.999	0.002	0.676	0.044	0.979
Anney et al., 2010 [30]	Autism	NA	rs6557675	A (minor allele)	0.61 (0.51–0.71)	2.20 × 10^−7^	0.000	0.126	0.006	0.861	0.000	0.001	0.000	0.048
Anney et al., 2012 [34]	Autism (European)	None	rs289858	A	0.52 (0.40–0.67)	2.81 × 10^−7^	0.000	0.027	0.762	1.000	0.015	0.940	0.161	0.995
Anney et al., 2012 [34]	ASD	SYNE2	rs2150291	A	1.72 (1.40–2.13)	2.83 × 10^−7^	0.000	0.105	0.579	0.999	0.006	0.864	0.119	0.993
Anney et al., 2012 [34]	ASD (European)	RPH3AL	rs7207517	A	1.97 (1.51–2.57)	3.05 × 10^−7^	0.000	0.022	0.817	1.000	0.025	0.963	0.226	0.997
Anney et al., 2012 [34]	Autism (European)	None	rs4761371	A	0.46 (0.34–0.63)	3.91 × 10^−7^	0.000	0.010	0.924	1.000	0.111	0.992	0.521	0.999
Anney et al., 2012 [34]	ASD (European)	PRAMEF12	rs1812242	A	1.44 (1.25–1.66)	4.29 × 10^−7^	0.006	0.713	0.077	0.988	0.001	0.411	0.038	0.975
Anney et al., 2012 [34]	ASD	None	rs10904487	G	0.63 (0.52–0.75)	4.29 × 10^−7^	0.001	0.262	0.198	0.996	0.001	0.440	0.028	0.966
Anney et al., 2012 [34]	Autism (European)	None	rs289932	A	0.67 (0.57–0.79)	5.42 × 10^−7^	0.005	0.524	0.286	0.998	0.004	0.784	0.135	0.994
Anney et al., 2010 [30]	Autism	MACROD2	rs4141463	A (minor allele)	0.62 (0.52–0.73)	5.50 × 10^−7^	0.000	0.192	0.047	0.980	0.000	0.048	0.002	0.655
Anney et al., 2012 [34]	Autism	None	rs9608521	A	1.46 (1.25–1.69)	7.62 × 10^−7^	0.004	0.641	0.084	0.989	0.001	0.383	0.033	0.971
Anney et al., 2012 [34]	ASD	None	rs1408744	A	0.65 (0.54–0.77)	8.06 × 10^−7^	0.002	0.385	0.235	0.997	0.002	0.618	0.062	0.985
Anney et al., 2017 [31]	ASD	LINC00535	chr8_94389815_I	I vs. D	1.14 (1.09–1.19)	9.47 × 10^−7^	0.990	1.000	0.000	0.002	0.000	0.002	0.686	1.000
Anney et al., 2012 [34]	ASD (European)	PC	rs7122539	A	0.60 (0.49–0.74)	9.64 × 10^−7^	0.001	0.162	0.628	0.999	0.011	0.917	0.213	0.996
Anney et al., 2010 [30]	Autism	MACROD2	rs4814324	A (minor allele)	1.58 (1.34–1.86)	9.80 × 10^−7^	0.000	0.266	0.076	0.988	0.000	0.128	0.006	0.859
Anney et al., 2010 [30]	Autism	MACROD2	rs6079544	A (minor allele)	1.57 (1.33–1.84)	1.20 × 10^−6^	0.000	0.287	0.053	0.982	0.000	0.081	0.004	0.797
Anney et al., 2017 [31]	ASD	EXOC4	rs6467494	T vs. C	1.12 (1.07–1.16)	1.43 × 10^−6^	1.000	1.000	0.000	0.000	0.000	0.000	0.197	0.996
Anney et al., 2010 [30]	Autism	MACROD2	rs6079536	A (minor allele)	0.64 (0.54–0.75)	1.60 × 10^−6^	0.001	0.307	0.059	0.984	0.000	0.102	0.005	0.837
Anney et al., 2010 [30]	ASD	MYH11	rs17284809	A (minor allele)	0.52 (0.39–0.69)	1.70 × 10^−6^	0.001	0.043	0.915	1.000	0.121	0.993	0.636	0.999
Anney et al., 2010 [30]	Autism	MACROD2	rs6079553	A (minor allele)	1.55 (1.31–1.82)	2.10 × 10^−6^	0.001	0.344	0.090	0.990	0.000	0.204	0.011	0.920
Anney et al., 2010 [30]	Autism	MACROD2	rs6074798	A (minor allele)	1.56 (1.32–1.84)	2.10 × 10^−6^	0.001	0.321	0.123	0.993	0.000	0.287	0.017	0.945
Anney et al., 2017 [31]	ASD	OPCML	rs7952100	C vs.G	1.14 (1.09–1.19)	2.49 × 10^−6^	0.990	1.000	0.000	0.002	0.000	0.002	0.686	1.000
Anney et al., 2010 [30]	Autism	MACROD2	rs10446030	G (minor allele)	1.54 (1.30–1.81)	3.20 × 10^−6^	0.001	0.375	0.116	0.992	0.000	0.301	0.019	0.951
Kuo et al., 2015 [33]	ASD	STYK1	rs16922945	C (minor allele)	1.86 (1.43–2.43)	3.43 × 10^−6^	0.001	0.057	0.891	1.000	0.085	0.989	0.572	0.999
Anney et al., 2010 [30]	ASD	POU5F2	rs10258862	G (minor allele)	1.41 (1.23–1.61)	3.70 × 10^−6^	0.009	0.820	0.043	0.978	0.000	0.319	0.027	0.966
Anney et al., 2010 [30]	Autism	MACROD2	rs6079540	A (minor allele)	0.65 (0.55–0.77)	3.70 × 10^−6^	0.002	0.385	0.235	0.997	0.002	0.618	0.062	0.985
Anney et al., 2010 [30]	Autism	MACROD2	rs6074787	A (minor allele)	1.53 (1.30–1.80)	4.10 × 10^−6^	0.002	0.406	0.147	0.994	0.001	0.418	0.031	0.970
Anney et al., 2010 [30]	ASD	MACROD2	rs6074798	A (minor allele)	1.38 (1.22–1.56)	4.80 × 10^−6^	0.013	0.909	0.020	0.954	0.000	0.224	0.018	0.948
Anney et al., 2010 [30]	Autism	MACROD2	rs980319	G (minor allele)	1.52 (1.29–1.79)	5.10 × 10^−6^	0.002	0.437	0.184	0.996	0.001	0.543	0.050	0.981
Anney et al., 2010 [30]	Autism	MACROD2	rs6079537	G (minor allele)	1.52 (1.29–1.79)	6.00 × 10^−6^	0.002	0.437	0.184	0.996	0.001	0.543	0.050	0.981
Kuo et al., 2015 [33]	ASD	NA	rs10966205	A (minor allele)	1.52 (1.27–1.83)	6.25 × 10^−6^	0.006	0.444	0.609	0.999	0.022	0.957	0.426	0.999
Kuo et al., 2015 [33]	ASD	OR2M4	rs10888329	T (minor allele)	0.55 (0.43–0.72)	8.05 × 10^−6^	0.001	0.081	0.916	1.000	0.144	0.994	0.718	1.000
Anney et al., 2010 [30]	ASD	MACROD2	rs6079536	A (minor allele)	0.73 (0.65–0.83)	8.50 × 10^−6^	0.022	0.917	0.067	0.986	0.002	0.628	0.084	0.989
Anney et al., 2010 [30]	ASD	NA	rs6557675	A (minor allele)	0.72 (0.63–0.82)	8.70 × 10^−6^	0.014	0.877	0.051	0.982	0.001	0.457	0.047	0.980
Kuo et al., 2015 [33]	ASD	NA	rs7933990	A (minor allele)	1.72 (1.35–2.19)	9.40 × 10^−6^	0.002	0.133	0.861	1.000	0.075	0.988	0.606	0.999
Kuo et al., 2015 [33]	ASD	MNT	rs2447097	A (minor allele)	1.53 (1.27–1.85)	9.45 × 10^−6^	0.006	0.419	0.652	0.999	0.026	0.965	0.467	0.999
Anney et al., 2010 [30]	ASD	GSG1L	rs205409	G (minor allele)	0.72 (0.64–0.82)	9.60 × 10^−6^	0.014	0.877	0.051	0.982	0.001	0.457	0.047	0.980
Kuo et al., 2015 [33]	ASD	OR2M4	rs6672981	C (minor allele)	0.55 (0.42–0.72)	9.64 × 10^−6^	0.001	0.081	0.916	1.000	0.144	0.994	0.718	1.000
Kuo et al., 2015 [33]	ASD	OR2M4	rs4397683	C (minor allele)	0.55 (0.42–0.72)	9.86 × 10^−6^	0.001	0.081	0.916	1.000	0.144	0.994	0.718	1.000
Anney et al., 2010 [30]	ASD	MACROD2	rs980319	G (minor allele)	1.36 (1.20–1.54)	1.00 × 10^−5^	0.024	0.939	0.049	0.981	0.001	0.570	0.068	0.987
Kuo et al., 2015 [33]	ASD	BCAS1/CYP24A1	rs12479663	G (minor allele)	1.81 (1.38–2.36)	1.08 × 10^−5^	0.001	0.083	0.907	1.000	0.124	0.993	0.687	1.000
Anney et al., 2010 [30]	ASD	MACROD2	rs4814324	A (minor allele)	1.36 (1.20–1.54)	1.10 × 10^−5^	0.024	0.939	0.049	0.981	0.001	0.570	0.068	0.987
Kuo et al., 2015 [33]	ASD	KRR1	rs3741496	C (minor allele)	1.49 (1.24–1.78)	1.15 × 10^−5^	0.009	0.529	0.565	0.999	0.020	0.954	0.430	0.999
Kuo et al., 2015 [33]	ASD	OR2M4	rs4642918	C (minor allele)	0.56 (0.43–0.73)	1.24 × 10^−5^	0.002	0.099	0.917	1.000	0.155	0.995	0.745	1.000
Anney et al., 2010 [30]	ASD	MACROD2	rs6079544	A (minor allele)	1.35 (1.20–1.53)	1.30 × 10^−5^	0.033	0.951	0.074	0.988	0.003	0.733	0.124	0.993
Kuo et al., 2015 [33]	ASD	NA	rs13211684	G (minor allele)	1.56 (1.28–1.91)	1.36 × 10^−5^	0.006	0.352	0.750	1.000	0.045	0.979	0.572	0.999
Kuo et al., 2015 [33]	ASD	MNT	rs2447095	A (minor allele)	1.52 (1.26–1.84)	1.45 × 10^−5^	0.008	0.446	0.695	1.000	0.038	0.975	0.552	0.999
Kuo et al., 2015 [33]	ASD	NA	rs12543592	G (minor allele)	0.67 (0.56–0.81)	1.63 × 10^−5^	0.012	0.521	0.744	1.000	0.063	0.985	0.678	1.000
Anney et al., 2010 [30]	ASD	MACROD2	rs6079553	A (minor allele)	1.35 (1.19–1.52)	1.70 × 10^−5^	0.026	0.959	0.027	0.965	0.001	0.424	0.041	0.977
Kuo et al., 2015 [33]	ASD	KRR1	rs1051446	C (minor allele)	1.47 (1.23–1.76)	1.77 × 10^−5^	0.014	0.587	0.669	1.000	0.045	0.979	0.614	0.999
Anney et al., 2010 [30]	ASD	NA	rs4078417	C (minor allele)	1.38 (1.21–1.57)	1.90 × 10^−5^	0.017	0.897	0.055	0.983	0.001	0.524	0.059	0.984
Anney et al., 2010 [30]	ASD	MACROD2	rs10446030	G (minor allele)	1.34 (1.19–1.52)	2.20 × 10^−5^	0.043	0.960	0.110	0.992	0.006	0.847	0.210	0.996
Kuo et al., 2015 [33]	ASD	GPD2	rs3916984	T (minor allele)	0.62 (0.49–0.77)	2.25 × 10^−5^	0.004	0.256	0.804	1.000	0.056	0.984	0.595	0.999
Kuo et al., 2015 [33]	ASD	NA	rs12366025	T (minor allele)	1.67 (1.31–2.11)	2.49 × 10^−5^	0.003	0.184	0.860	1.000	0.086	0.989	0.662	0.999
Ma et al., 2009 [32]	Autism	NA	rs10038113	C(minor)/T(major)	0.67 (0.56–0.81)	2.75 × 10^−5^	0.012	0.521	0.744	1.000	0.063	0.985	0.678	1.000
Anney et al., 2010 [30]	ASD	MACROD2	rs6079540	A (minor allele)	0.75 (0.66–0.84)	2.90 × 10^−5^	0.034	0.979	0.019	0.950	0.001	0.399	0.037	0.975
Anney et al., 2010 [30]	Autism	HAT1	rs6731562	G (minor allele)	1.51 (1.27–1.81)	3.30 × 10^−5^	0.006	0.471	0.562	0.999	0.017	0.946	0.383	0.998
Anney et al., 2010 [30]	ASD	MACROD2	rs6074787	A (minor allele)	1.33 (1.18–1.50)	3.40 × 10^−5^	0.047	0.975	0.067	0.986	0.003	0.776	0.147	0.994
Kuo et al., 2015 [33]	ASD	GLIS1	rs12080933	A (minor allele)	1.48 (1.23–1.78)	3.57 × 10^−5^	0.013	0.557	0.707	1.000	0.053	0.983	0.648	0.999
Kuo et al., 2015 [33]	ASD	FSTL4	rs11741756	T (minor allele)	1.67 (1.31–2.13)	3.64 × 10^−5^	0.004	0.194	0.903	1.000	0.157	0.995	0.785	1.000
Kuo et al., 2015 [33]	ASD	STYK1	rs7953930	G (minor allele)	1.65 (1.30–2.09)	3.83 × 10^−5^	0.004	0.215	0.888	1.000	0.133	0.994	0.761	1.000
Anney et al., 2010 [30]	Autism	NA	rs4078417	C (minor allele)	1.50 (1.26–1.79)	4.10 × 10^−5^	0.007	0.500	0.509	0.999	0.014	0.933	0.339	0.998
Anney et al., 2010 [30]	ASD	MACROD2	rs4141463	A (minor allele)	0.75 (0.66–0.85)	4.30 × 10^−5^	0.049	0.967	0.118	0.993	0.007	0.873	0.243	0.997
Kuo et al., 2015 [33]	ASD	OR2M3	rs11204613	G (minor allele)	0.58 (0.45–0.75)	4.60 × 10^−5^	0.003	0.144	0.920	1.000	0.185	0.996	0.799	1.000
Anney et al., 2010 [30]	ASD	MACROD2	rs6079537	G (minor allele)	1.32 (1.17–1.49)	5.40 × 10^−5^	0.062	0.981	0.103	0.991	0.007	0.878	0.249	0.997
Anney et al., 2010 [30]	Autism	GSG1L	rs205409	G (minor allele)	0.69 (0.58–0.81)	1.10 × 10^−4^	0.011	0.663	0.353	0.998	0.009	0.896	0.271	0.997
Anney et al., 2010 [30]	Autism	POU5F2	rs10258862	G (minor allele)	1.43 (1.21–1.71)	1.80 × 10^−4^	0.027	0.700	0.764	1.000	0.112	0.992	0.799	1.000

Abbreviations: ASD, Autism spectrum disorders; A, Adenine; C, Cytosine; G, Guanine; T, Thymine; D, Deletion; I, Insertion; FPRP, false positive rate probability; BFDP, Bayesian false discovery probability; OR, odds ratio; CI, confidence interval; GWAS, Genome-Wide Association Studies; NA, not available.

**Table 5:** Lists of genes involved in the PPI network.

Gene	Function of the Encoding Proteins
OXTR	Receptor for oxytocin associated with social recognition and emotion processing
MTHFR	Influences susceptibility to neural tube defect by changing folate metabolism
RELN	Control cell positioning and neural migration during brain development
DRD3	D3 subtype of the five dopamine receptors; localized to the limbic areas of the brain
MNT	Protein member of the Myc/Max/Mad network; transcriptional repressor and an antagonist of Myc-dependent transcriptional activation and cell growth
OPCML	Member of the IgLON subfamily in the immunoglobulin protein superfamily of proteins; localized in the plasma membrane; accessory role in opioid receptor function
PC	Pyruvate carboxylase; gluconeogenesis, lipogenesis, insulin secretion and synthesis of neurotransmitter glutamate
ERBB4	Tyr protein kinase family and the epidermal growth factor receptor subfamily; binds to and is activated by neuregulins, and induces mitogenesis and differentiation
OR2M4	Members of a large family of GPCR; olfactory receptors initiating a neuronal response that triggers the perception of a smell
BCAS1	Oncogene; highly expressed in three amplified breast cancer cell lines and in one breast tumor without amplification at 20q13.2.
CYP24A1	Cytochrome P450 superfamily of enzymes; drug metabolism and synthesis of cholesterol, steroids and other lipids
TMEM132B	The function remains poorly understood despite their mutations associated with non-syndromic hearing loss, panic disorder, and cancer
KRR1	Nucleolar protein; 18S rRNA synthesis and 40S ribosomal assembly
HAT1	Type B histone acetyltransferase; rapid acetylation of newly synthesized cytoplasmic histones; replication-dependent chromatin assembly
SGSM2	GTPase activator; regulators of membrane trafficking
EXT1	Endoplasmic reticulum-resident type II transmembrane glycosyltransferase; involved in the chain elongation step of heparan sulfate biosynthesis
OR2T33	Members of a large family of GPCR; share a 7-transmembrane domain structure with many neurotransmitter and hormone receptors
TAF1C	Binds to the core promoter of ribosomal RNA genes to position the polymerase properly; acts as a channel for regulatory signals
HDAC4	Class II of the histone deacetylase/acuc/apha family; represses transcription when tethered to a promoter
MEGF10	Member of the multiple epidermal growth factor-like domains protein family; cell adhesion, motility and proliferation; critical mediator of apoptotic cell phagocytosis; amyloid-beta peptide uptake in brain
NFKB2	Subunit of the transcription factor complex nuclear factor-kappa-B; central activator of genes involved in inflammation and immune function
BNC2	Conserved zinc finger protein; skin color saturation
NMB	Member of the bombesin-like family of neuropeptides; negatively regulate eating behavior; regulate colonic smooth muscle contraction
HPS6	Organelle biogenesis associated with melanosomes, platelet dense granules, and lysosomes
ELOVL3	GNS1/SUR4 family; elongation of long chain fatty acids to provide precursors for synthesis of sphingolipids and ceramides
PITX3	Member of the RIEG/PITX homeobox family; transcription factors; lens formation during eye development
NAALADL2	Not well-known, but diseases associated with NAALADL2 include Chromosome 6Pter-P24 Deletion Syndrome and Cornelia De Lange Syndrome.
MACROD2	Deacetylase removing ADP-ribose from mono-ADP-ribosylated proteins; translocate from the nucleus to the cytoplasm upon DNA damage
CUEDC2	CUE domain-containing protein; down-regulate ESR1 protein levels through progesterone-induced and degradation of receptors
FBXL15	Substrate recognition component of SCF E3 ubiquitin-protein ligase complex; mediates the ubiquitination and subsequent proteasomal degradation of SMURF1
EXOC4	Component of the exocyst complex; targeting exocytic vesicles to specific docking sites on the plasma membrane
NOLC1	Nucleolar protein; act as a regulator of RNA polymerase I; neural crest specification; nucleologenesis
PPRC1	Similar to PPAR-gamma coactivator 1; activate mitochondrial biogenesis through NRF1 in response to proliferative signals
SEC11A	Member of the peptidase S26B family; subunit of the signal peptidase complex; cell migration and invasion, gastric cancer and lymph node metastasis

Abbreviations: OXTR, Oxytocin Receptor; MTHFR, Methylene tetrahydrofolate reductase; RELN, reelin, DRD3, Dopamine Receptor D3; MNT, Myc-associated factor X (MAX) Network Transcriptional Repressor; OPCML, opioid binding protein/cell adhesion molecule-like; PC, Pyruvate carboxylase; ERBB4, Erb-B2 Receptor Tyrosine Kinase 4; OR2M4, olfactory receptor family 2 subfamily M member 4; GPCR, G protein-coupled receptor; BCAS1, Breast Carcinoma Amplified Sequence 1; CYP24A1, Cytochrome P450 Family 24 Subfamily A Member 1; TMEM132B, transmembrane protein 132B; KRR1, KRR1 small subunit processome component homolog; HAT1, histone acetyltransferase 1; SGSM2, small G protein signaling modulator 2; EXT1, Exostosin-1; OR2T33, Olfactory receptor 2T33; TAF1C, TATA-Box Binding Protein Associated Factor, RNA Polymerase I Subunit C; HDAC4, Histone deacetylase 4; MEGF10, Multiple Epidermal Growth Factor Like Domains 10; NFKB2, Nuclear Factor Kappa B Subunit 2; BNC2, basonuclin-2; NMB, Neuromedin B; HPS6, Hermansky–Pudlak syndrome 6; ELOVL3, Elongation Of Very Long Chain Fatty Acids Protein 3, PITX3, Pituitary homeobox 3; NAALADL2, N-Acetylated Alpha-Linked Acidic Dipeptidase Like 2; MACROD2, Mono-ADP Ribosylhydrolase 2; CUEDC2, CUE domain containing 2; FBXL15, F-Box And Leucine Rich Repeat Protein 15; EXOC4, Exocyst Complex Component 4; NOLC1, Nucleolar And Coiled-Body Phosphoprotein 1; PPRC1, peroxisome proliferator-activated receptor gamma, coactivator-related 1; SEC11A, SEC11 Homolog A, Signal Peptidase Complex Subunit.

## Supplementary Materials

The following are available online at https://www.mdpi.com/2076-3425/10/10/692/s1, Supplementary Table S1. PRISMA 2009 Checklist; Supplementary Table S2. Gene variants without statistical significance (*p*-value ≥ 0.05) in meta-analyses of observational studies; Supplementary Table S3. Non-re-analyzable gene variants with genome wide statistical significance (*p*-value < 5 × 10^−8^) from the GWAS catalog, meta-analyses of GWAS and the GWAS datasets included in the GWAS meta-analysis.
