# Supplementary material for: Genetic Variation and Autism: A Field Synopsis and Systematic Meta-Analysis
Source: Brain Sci. 2020 Sep 30;10(10):692. doi: 10.3390/brainsci10100692 (PMC7600188; doi:10.3390/brainsci10100692)
Supplement: Supplementary file 1 [file brainsci-10-00692-s001.zip › Suppl/brainsci-920670_SPL_RV.docx]

**Supplements**

Genetic variation in autism spectrum disorder: A field synopsis of systematic meta-analysis

**Supplementary Table S1.** PRISMA 2009 Checklist

| **Section/topic** | **#** | **Checklist item** | **Reported on page #** |
| --- | --- | --- | --- |
| **TITLE** | | |  |
| Title | 1 | Identify the report as a systematic review, meta-analysis, or both. | 1 |
| **ABSTRACT** | | |  |
| Structured summary | 2 | Provide a structured summary including, as applicable: background; objectives; data sources; study eligibility criteria, participants, and interventions; study appraisal and synthesis methods; results; limitations; conclusions and implications of key findings; systematic review registration number. | 2 |
| **INTRODUCTION** | | |  |
| Rationale | 3 | Describe the rationale for the review in the context of what is already known. | 3 |
| Objectives | 4 | Provide an explicit statement of questions being addressed with reference to participants, interventions, comparisons, outcomes, and study design (PICOS). | 3 |
| **METHODS** | | |  |
| Protocol and registration | 5 | Indicate if a review protocol exists, if and where it can be accessed (e.g., Web address), and, if available, provide registration information including registration number. | 2 (PROSPERO,  CRD42018091704) |
| Eligibility criteria | 6 | Specify study characteristics (e.g., PICOS, length of follow-up) and report characteristics (e.g., years considered, language, publication status) used as criteria for eligibility, giving rationale. | 4 |
| Information sources | 7 | Describe all information sources (e.g., databases with dates of coverage, contact with study authors to identify additional studies) in the search and date last searched. | 4 |
| Search | 8 | Present full electronic search strategy for at least one database, including any limits used, such that it could be repeated. | 4 (figure 2) |
| Study selection | 9 | State the process for selecting studies (i.e., screening, eligibility, included in systematic review, and, if applicable, included in the meta-analysis). | 4 |
| Data collection process | 10 | Describe method of data extraction from reports (e.g., piloted forms, independently, in duplicate) and any processes for obtaining and confirming data from investigators. | 4 |
| Data items | 11 | List and define all variables for which data were sought (e.g., PICOS, funding sources) and any assumptions and simplifications made. | 4-5 |
| Risk of bias in individual studies | 12 | Describe methods used for assessing risk of bias of individual studies (including specification of whether this was done at the study or outcome level), and how this information is to be used in any data synthesis. | 4-5 |
| Summary measures | 13 | State the principal summary measures (e.g., risk ratio, difference in means). | 4-5, tables and supplementary tables |
| Synthesis of results | 14 | Describe the methods of handling data and combining results of studies, if done, including measures of consistency (e.g., I^2^) for each meta-analysis. | 4-5, supplementary description of criteria for classification of levels of evidence |

| **Section/topic** | **#** | **Checklist item** | **Reported on page #** |
| --- | --- | --- | --- |
| Risk of bias across studies | 15 | Specify any assessment of risk of bias that may affect the cumulative evidence (e.g., publication bias, selective reporting within studies). | 4-5 |
| Additional analyses | 16 | Describe methods of additional analyses (e.g., sensitivity or subgroup analyses, meta-regression), if done, indicating which were pre-specified. | Tables and supplementary tables |
| **RESULTS** | | |  |
| Study selection | 17 | Give numbers of studies screened, assessed for eligibility, and included in the review, with reasons for exclusions at each stage, ideally with a flow diagram. | Figure 2 |
| Study characteristics | 18 | For each study, present characteristics for which data were extracted (e.g., study size, PICOS, follow-up period) and provide the citations. | 6-7, tables and supplementary tables |
| Risk of bias within studies | 19 | Present data on risk of bias of each study and, if available, any outcome level assessment (see item 12). | Tables and supplementary tables |
| Results of individual studies | 20 | For all outcomes considered (benefits or harms), present, for each study: (a) simple summary data for each intervention group (b) effect estimates and confidence intervals, ideally with a forest plot. | 6-7, tables and supplementary tables |
| Synthesis of results | 21 | Present results of each meta-analysis done, including confidence intervals and measures of consistency. | Tables and supplementary tables |
| Risk of bias across studies | 22 | Present results of any assessment of risk of bias across studies (see Item 15). | Tables and supplementary tables |
| Additional analysis | 23 | Give results of additional analyses, if done (e.g., sensitivity or subgroup analyses, meta-regression [see Item 16]). | Tables and supplementary tables |
| **DISCUSSION** | | |  |
| Summary of evidence | 24 | Summarize the main findings including the strength of evidence for each main outcome; consider their relevance to key groups (e.g., healthcare providers, users, and policy makers). | 8-9, tables and supplementary tables |
| Limitations | 25 | Discuss limitations at study and outcome level (e.g., risk of bias), and at review-level (e.g., incomplete retrieval of identified research, reporting bias). | 8-9 |
| Conclusions | 26 | Provide a general interpretation of the results in the context of other evidence, and implications for future research. | 9 |
| **FUNDING** | | |  |
| Funding | 27 | Describe sources of funding for the systematic review and other support (e.g., supply of data); role of funders for the systematic review. | 2 |

*From:*  Moher D, Liberati A, Tetzlaff J, Altman DG, The PRISMA Group (2009). Preferred Reporting Items for Systematic Reviews and Meta-Analyses: The PRISMA Statement. PLoS Med 6(7): e1000097. doi:10.1371/journal.pmed1000097.

**Supplementary Table S2. Gene variants without statistical significance (*P*-value≥0.05) in meta-analyses of observational studies**

| **Author, Year** | **Gene/Variant** | **Comparison** | **OR (95% CI)** | **P-value** | **Model** | **No. of studies** | |
| --- | --- | --- | --- | --- | --- | --- | --- |
|  |  |  |  |  |  |  |  |
|  |  |  |  |  |  |  |  |
| Main et al., 2012 | MTHFR/C677T | TT vs. CC | 2.26 (1.30, 3.91) | NA | Random | Overall6 | |
| Huang et al., 2008 | SLC6A4/5-HTTLPR | Short vs. Long | 1.045 (0.878, 1.245) | NA | NA | Overall14 | |
| Curran et al., 2011 | MACROD2/rs4141463 in ASD | C vs. T | 1.034 (0.944, 1.133) | 0.5 | NA | European5 | |
| Curran et al., 2011 | MACROD2/rs4141463 in ausitm | C vs. T | 0.986 (0.88, 1.11) | 0.85 | NA | European5 | |
| Yang et al., 2017 | SLC6A4/5-HTTLPR | S vs. L | 1.05 (0.92, 1.20) | 0.4652 | Random | Overall22 | |
| Yang et al., 2017 | SLC6A4/5-HTTLPR | S vs. L | 1.18883(0.85879, 1.6457) | 0.2972 | Random | Overall6 | |
| Yang et al., 2017 | COMT/rs4680 | Met vs. Val | 1.18 (0.87, 1.59) | 0.29 | Random | Overall5 | |
| Yang et al., 2017 | COMT/rs4680 | Met vs. Val | 1.67487(0.82263,3.41003) | 0.15511 | Random | Overall2 | |
| Warrier et al., 2015 | STX1A/rs6951030 | G vs. T | 1.383 (0.995, 1.922) | 5.40×10^-2^ | Random | Overall3 | |
| Warrier et al., 2015 | OXTR/rs2268493 | C vs. T | 0.845 (0.701, 1.019) | 7.80×10^-2^ | Fixed | Overall3 | |
| Warrier et al., 2015 | ASMT/rs5989681 | G vs. C | 1.135 (0.984, 1.308) | 8.20×10^-2^ | Fixed | Overall3 | |
| Warrier et al., 2015 | RELN/rs2073559 | C vs. T | 0.955 (0.900, 1.014) | 1.35×10^-1^ | Fixed | Overall3 | |
| Warrier et al., 2015 | GLO1/rs2736654 | A vs. C | 1.307 (0.882, 1.936) | 1.82×10^-1^ | Random | Overall4 | |
| Warrier et al., 2015 | PON1/rs854560 | A vs. T | 1.140 (0.931, 1.395) | 2.05×10^-1^ | Fixed | Overall2 | |
| Warrier et al., 2015 | TPH2/rs11179000 | T vs. A | 1.130 (0.934, 1.366) | 2.08×10^-1^ | Fixed | Overall3 | |
| Warrier et al., 2015 | MET/rs1858830 | G vs. C | 0.905 (0.773, 1.061) | 2.19×10^-1^ | Random | Overall6 | |
| Warrier et al., 2015 | MET/rs1858830 | G vs. C | 0.889 (0.749, 1.056) | 0.180 | Random | CC4 | |
| Warrier et al., 2015 | OXTR/rs2268490 | T vs. C | 1.135 (0.920, 1.400) | 2.38×10^-1^ | Fixed | Overall3 | |
| Warrier et al., 2015 | OXTR/rs2301261 | A vs. G | 1.127 (0.889, 1.430) | 3.22×10^-1^ | Fixed | Overall4 | |
| Warrier et al., 2015 | HOXA1/rs10951154 | G vs. A | 0.925 (0.791, 1.081) | 3.28×10^-1^ | Fixed | Overall13 | |
| Warrier et al., 2015 | BDNF/rs6265 | G vs. A | 0.919 (0.763, 1.107) | 3.72×10^-1^ | Fixed | Overall3 | |
| Warrier et al., 2015 | HTR2A/rs6311 | A vs. G | 0.871 (0.643, 1.181) | 3.74×10^-1^ | Random | Overall6 | |
| Warrier et al., 2015 | HTR2A/rs6311 | A vs. G | 0.893 (0.602, 1.325) | 0.573 | Random | TDT4 | |
| Warrier et al., 2015 | ITGB3/rs5918 | C vs. T | 0.866 (0.630, 1.191) | 3.77×10^-1^ | Fixed | Overall3 | |
| Warrier et al., 2015 | MAOA/uVNTR | short vs. long | 0.781 (0.446, 1.368) | 3.87×10^-1^ | Random | Overall3 | |
| Warrier et al., 2015 | MACROD2/rs4141463 | T vs. C | 0.913 (0.734, 1.135) | 4.11×10^-1^ | Random | Overall3 | |
| Warrier et al., 2015 | OXTR/rs2254298 | A vs. G | 0.813 (0.489, 1.352) | 4.25×10^-1^ | Random | Overall5 | |
| Warrier et al., 2015 | OXTR/rs2254298 | A vs. G | 0.664 (0.357, 1.235) | 0.196 | Fixed | Caucasian4 | |
| Warrier et al., 2015 | ASMT/rs6644635 | C vs. T | 1.056 (0.906, 1.230) | 4.88×10^-1^ | Fixed | Overall4 | |
| Warrier et al., 2015 | SLC6A4/rs2020942 | A vs. G | 1.062 (0.881, 1.281) | 5.28×10^-1^ | Fixed | Overall3 | |
| Warrier et al., 2015 | OMG/rs11080149 | A vs. G | 0.847 (0.477, 1.506) | 5.72×10^-1^ | Random | Overall2 | |
| Warrier et al., 2015 | ADA/rs7359837 | G vs. A | 1.375 (0.401, 4.717) | 6.13×10^-1^ | Random | Overall3 | |
| Warrier et al., 2015 | OXTR/rs237894 | G vs. C | 0.961 (0.818, 1.129) | 6.26×10^-1^ | Fixed | Overall3 | |
| Warrier et al., 2015 | OXTR/rs53576 | A vs. G | 0.966 (0.839, 1.113) | 6.31×10^-1^ | Fixed | Overall5 | |
| Warrier et al., 2015 | OXTR/rs2268494 | A vs. T | 1.076 (0.760, 1.510) | 6.73×10^-1^ | Fixed | Overall2 | |
| Warrier et al., 2015 | SLC6A4/STin2 VNTR | 12 vs. 9/10 | 1.129 (0.819, 1.558) | 6.73×10^-1^ | Random | Overall8 | |
| Warrier et al., 2015 | NF1/GxAlu | 9 vs. non-9 | 1.131 (0.633, 2.022) | 6.77×10^-1^ | Random | Overall3 | |
| Warrier et al., 2015 | GRIK2/rs2227281 | T vs. C | 0.929 (0.603, 1.432) | 7.32×10^-1^ | Random | Overall4 | |
| Warrier et al., 2015 | OXTR/rs2268495 | A vs. G | 1.059 (0.763, 1.468) | 7.33×10^-1^ | Fixed | Overall2 | |
| Warrier et al., 2015 | SHANK3/rs9616915 | C vs. T | 0.974 (0.834, 1.138) | 7.44×10^-1^ | Fixed | Overall3 | |
| Warrier et al., 2015 | HTR2A/rs6314 | T vs. G | 0.949 (0.691, 1.304) | 7.47×10^-1^ | Fixed | Overall4 | |
| Warrier et al., 2015 | CNTNAP2/rs2710102 | T vs. C | 0.989 (0.924, 1.059) | 7.60×10^-1^ | Fixed | Overall2 | |
| Warrier et al., 2015 | OXTR/rs237885 | G vs. T | 0.981 (0.868, 1.109) | 7.62×10^-1^ | Fixed | Overall4 | |
| Warrier et al., 2015 | OXTR/rs237885 | G vs. T | 0.950 (0.817, 1.106) | 0.511 | Fixed | CC3 | |
| Warrier et al., 2015 | COMT/rs4680 | A vs. G | 0.982 (0.851, 1.134) | 8.08×10^-1^ | Fixed | Overall5 | |
| Warrier et al., 2015 | COMT/rs4680 | A vs. G | 0.973 (0.840, 1.128) | 0.717 | Fixed | TDT2 | |
| Warrier et al., 2015 | MTHFR/rs1801131 | C vs. A | 0.979 (0.824, 1.164) | 8.11×10^-1^ | Random | Overall3 | |
| Warrier et al., 2015 | MTHFR/rs1801131 | C vs. A | 0.904 (0.782, 1.044) | 0.171 | Fixed | Caucasian3 | |
| Warrier et al., 2015 | OXTR/rs1042778 | G vs. A | 1.02 (0.849, 1.225) | 8.33×10^-1^ | Fixed | Overall2 | |
| Warrier et al., 2015 | GRIK2/rs2227283 | A vs. G | 0.967 (0.686, 1.363) | 8.51×10^-1^ | Random | Overall4 | |
| Warrier et al., 2015 | EN2/rs3735653 | T vs. C | 1.007 (0.870, 1.165) | 9.28×10^-1^ | Fixed | Overall4 | |
| Warrier et al., 2015 | NF1/GxAlu | 8 vs. non-8 | 0.982 (0.602, 1.601) | 9.41×10^-1^ | Random | Overall3 | |
| Warrier et al., 2015 | SLC6A4/5-HTTLPR | short vs. long | 0.994 (0.847, 1.167) | 9.42×10^-1^ | Random | Overall16 | |
| Warrier et al., 2015 | SLC6A4/5-HTTLPR | short vs. long | 0.960 (0.650, 1.418) | 0.838 | Fixed | Caucasian5 | |
| Warrier et al., 2015 | HTR2A/rs6313 | T vs. C | 1.007 (0.812, 1.249) | 9.47×10^-1^ | Fixed | NA | |
| Warrier et al., 2015 | EN2/rs1861973 | T vs. C | 1.004 (0.775, 1.300) | 9.77×10^-1^ | Random | Overall5 | |
| Song et al., 2011 | HOXA1/rs10951154 | G vs. A | 0.97 (0.76, 1.31) | 0.843 | Random | Overall10 | |
| Song et al., 2011 | HOXA1/rs10951154 | G vs. A | 0.98 (0.74, 1.34) | 0.900 | Random | TDT8 | |
| Song et al., 2011 | HOXA1/rs10951154 | G vs. A | 0.92 (0.67, 1.26) | 0.588 | Random | CC2 | |
| Song et al., 2011 | HOXB1/rs72338773 | INS vs. nINS | 1.14 (0.97, 1.33) | 0.118 | Fixed | Overall7 | |
| Song et al., 2011 | HOXB1/rs72338773 | INS vs. nINS | 1.16 (0.98, 1.38) | 0.079 | Fixed | TDT5 | |
| Song et al., 2011 | HOXB1/rs72338773 | INS vs. nINS | 1.13 (0.90, 1.42) | 0.284 | Fixed | CC2 | |
| Chen et al., 2017 | RELN/rs736707 | C (Additive.) | 0.90 (0.67, 1.20) | 0.47 | Fixed | Overall5 | |
| Chen et al., 2017 | RELN/rs736707 | C (Dominant.) | 0.87 (0.57, 1.33) | 0.53 | Fixed | Overall5 | |
| Chen et al., 2017 | RELN/rs362691 | C (Additive.) | 0.88 (0.70, 1.10) | 0.26 | Fixed | Overall5 | |
| Chen et al., 2017 | RELN/rs362691 | C (Dominant.) | 0.87 (0.68, 1.11) | 0.25 | Fixed | Overall5 | |
| Chen et al., 2017 | RELN/rs607755 | A (Additive.) | 0.73 (0.53, 1.02) | 0.07 | Random | Overall3 | |
| Chen et al., 2017 | RELN/rs607755 | A (Dominant.) | 0.76 (0.48, 1.20) | 0.24 | Fixed | Overall3 | |
| Chen et al., 2017 | RELN/rs2229864 | T (Additive.) | 1.01 (0.83, 1.24) | 0.89 | Fixed | Overall4 | |
| Chen et al., 2017 | RELN/rs2229864 | T (Dominant.) | 1.08 (0.84, 1.38) | 0.55 | Fixed | Overall4 | |
| LoParo et al., 2015 | OXTR/rs1042778 | T (minor allele) | 0.97 (0.87, 1.09) | 0.6495 | Random | Overall(6)^a^ | |
| LoParo et al., 2015 | OXTR/rs237885 | G (minor allele) | 0.96 (0.85, 1.08) | 0.4583 | Random | Overall(8)^a^ | |
| LoParo et al., 2015 | OXTR/rs11706648 | C (minor allele) | 1.02 (0.89, 1.18) | 0.7552 | Random | Overall(4)^a^ | |
| LoParo et al., 2015 | OXTR/rs2268490 | T (minor allele) | 1.13 (0.93, 1.34) | 0.2000 | Random | Overall(4)^a^ | |
| LoParo et al., 2015 | OXTR/rs237888 | C (minor allele) | 1.17 (0.92, 1.50) | 0.2051 | Random | Overall(4)^a^ | |
| LoParo et al., 2015 | OXTR/rs4686301 | T (minor allele) | 1.15 (0.92, 1.43) | 0.2157 | Random | Overall(4)^a^ | |
| LoParo et al., 2015 | OXTR/rs2268493 | C (minor allele) | 0.98 (0.71, 1.33) | 0.8776 | Random | Overall(4)^a^ | |
| LoParo et al., 2015 | OXTR/rs2254298 | A (minor allele) | 1.15 (0.93, 1.43) | 0.1646 | Random | Overall5 | |
| LoParo et al., 2015 | OXTR/rs53576 | A (minor allele) | 0.91 (0.76, 1.09) | 0.3162 | Random | Overall(5)^a^ | |
| LoParo et al., 2015 | OXTR/rs237894 | C (minor allele) | 1.03 (0.84, 1.27) | 0.7759 | Random | Overall(4)^a^ | |
| LoParo et al., 2015 | OXTR/rs237895 | T (minor allele) | 1.21 (0.98, 1.48) | 0.0724 | Random | Overall(4)^a^ | |
| LoParo et al., 2015 | OXTR/rs2268495 | A (minor allele) | 0.97 (0.78, 1.21) | 0.7921 | Random | Overall(6)^a^ | |
| LoParo et al., 2015 | OXTR/rs4684302 | T (minor allele) | 0.87 (0.64, 1.23) | 0.4687 | Random | Overall(4)^a^ | |
| Wang et al., 2014 | RELN/rs736707 | R vs. NR | 1.11 (0.80, 1.54) | 0.546 | Random | Overall6 | |
| Wang et al., 2014 | RELN/rs736707 | R vs. NR | 1.19 (0.92, 1.54) | 0.175 | Fixed | TDT2 | |
| Wang et al., 2014 | RELN/GGC repeat | R vs. NR | 1.09 (0.97, 1.23) | 0.153 | Fixed | Overall7 | |
| Wang et al., 2014 | RELN/GGC repeat | R vs. NR | 1.08 (0.94, 1.24) | 0.281 | Fixed | TDT5 | |
| Kranz et al., 2016 | OXTR/rs237897 | T vs. C | 1.05 (0.88, 1.25) | 0.594 | Random | Overall3 | |
| Werling et al., 2016 | CNTNAP2/rs2710102 | NA | 1.028 (0.98, 1.08) | 0.26 | Fixed | Overall5 | |
| Werling et al., 2016 | CNTNAP2/rs7794745 | T | 1.023 (0.99, 1.05) | 0.112 | Fixed | Overall6 | |
| Noroozi et al, 2018 | GABRB3/rs20317 | CG+GG vs. CC | 0.97 (0.65, 1.44) | NA | Random | Overall3 | |
| Noroozi et al, 2018 | GABRB3/rs4906902 | AG+GG vs. AA | 0.97 (0.81, 1.17) | NA | Random | Overall5 | |
| Mahdavi et al, 2018 | GABRB3/rs2081648 | NA | 0.84(0.41, 1.72) | NA | Random | Overall4 | |
| Mahdavi et al, 2018 | GABRB3/rs1426217 | NA | 1.13(0.64, 2.00) | NA | Random | Overall3 | |
| Abbreviations: OR, odds ratio; CI, confidence interval; NA, not available; VNTR, variable number of tandem repeats; R, risk allele; NR, non-risk allele; A, Adenine; C, Cytosine; G; Guanine; T, Thymine; Additive, additive model; Dominant, dominant model; TDT, transmission disequilibrium test; CC, case-control study; INS, insertion; nINR, non-insertion.  a This article reported only the number of datasets not the number of individual studies included in the meta-analysis of this SNP. Thus, we wrote the number of datasets in the parenthesis. | | | | | | |  |

**Supplementary Table S3.** Non-re-analyzable gene variants with genome wide statistical significance (*p*-value<5×10^-8^) from the GWAS catalog, meta-analyses of GWAS and the GWAS datasets included in the GWAS meta-analysis

| **Author, Year** | **Reported gene(s)** | **Mapped gene(s)** | **Variant** | **Comparison** | **P-value** |
| --- | --- | --- | --- | --- | --- |
| Wang et al., 2009 (Catalog) | CDH10, CDH9 | MSNP1 – RNU4-43P | rs4307059 | T | 2.00E-10 |
| Wang et al., 2009 (GWAS meta) | NA | NA | rs4307059 | C (minor)/ T (major) | 2.10E-10 |
| Wang et al., 2009 (GWAS meta) | NA | NA | rs7704909 | C (minor)/ T (major) | 9.90E-10 |
| Wang et al., 2009 (GWAS meta) | NA | NA | rs12518194 | G (minor)/ A (major) | 1.10E-09 |
| Wang et al., 2009 (GWAS meta) | NA | NA | rs4327572 | T (minor)/ C (major) | 2.70E-09 |
| Wang et al., 2009 (GWAS) | NA | NA | rs4307059 | C (minor)/ T (major) | 3.40E-08 |
| Wang et al., 2009 (GWAS meta) | NA | NA | rs1896731 | C (minor)/ T (major) | 4.80E-08 |
| Xia et al., 2014 (Catalog) | TRIM33, BCAS2, DENND2C | TRIM33 | rs6537825 | A | 3.00E-08 |
| Xia et al., 2014 (Catalog) | AMPD1, NRAS, CASDE1 | CSDE1; AMPD1 - RN7SL432P | rs8453; rs926938 | G; A | 3.00E-08 |
| Xia et al, 2014 (GWAS meta) | TRIM33 | NA | rs6537835 | A (minor allele) | 3.26E-08 |
| Xia et al., 2014 (Catalog) | AMPD1, NRAS, CSDE1 | AMPD1 - RN7SL432P | rs926938 | A | 4.00E-08 |
| Xia et al., 2014 (Catalog) | TRIM33 | TRIM33; TRIM33; TRIM33 - PKMP1; TRIM33; TRIM33;  TRIM33; TRIM33; TRIM33; TRIM33 | rs3827735; rs11582563; rs11102807; rs7511633; rs11102800;  rs6537825; rs11585926; rs6661053; rs11589568 | C; G; A; G; C; A; T; T; T | 4.00E-08 |
| Xia et al, 2014 (GWAS meta) | AMPD1-NRAS | NA | rs926938 | A (minor allele) | 4.49E-08 |
| Grove et al. 2019 (catalog) | LOCI102723661m PTPB2 | RNU1 – 130P, AC099566.1 | rs201910565 | T (minor allele) | 4.00E-08 |
| Abbreviations: NA, not available; A, Adenine; C, Cytosine; G; Guanine; T, Thymine. | | | | | |
